# Hypoxic Jumbo Spheroids On-A-Chip (HOnAChip): Insights into Treatment Efficacy

**DOI:** 10.3390/cancers13164046

**Published:** 2021-08-11

**Authors:** Elena Refet-Mollof, Ouafa Najyb, Rodin Chermat, Audrey Glory, Julie Lafontaine, Philip Wong, Thomas Gervais

**Affiliations:** 1 Insitute of Biomedical Engineering, Polytechnique Montréal, 2500 Chemin de Polytechnique, Montréal, QC H3T 1J4, Canada; elena.refet@polymtl.ca (E.R.-M.); rodin.chermat@polymtl.ca (R.C.); 2 Institut du Cancer de Montréal, (ICM), Centre de Recherche du Centre Hospitalier de l’Université de Montréal (CRCHUM), 900 St. Denis Street, Montréal, QC H2X 0A9, Canada; ouafa.najyb.chum@ssss.gouv.qc.ca (O.N.); audrey.glory.chum@ssss.gouv.qc.ca (A.G.); julie.lafontaine.chum@ssss.gouv.qc.ca (J.L.); 3 Department of Radiation Oncology, Centre Hospitalier de l’Université de Montréal (CHUM), 1051 Sanguinet Street, Montréal, QC H2X 3E4, Canada; 4 Department of Radiation Oncology, Princess Margaret Cancer Centre, 610 University Avenue, Toronto, ON M5G 2M9, Canada; 5 Department of Radiation Oncology, University of Toronto, 149 College Street, Suite 504, Toronto, ON M5T 1P5, Canada; 6 Department of Engineering Physics, Polytechnique Montréal, 2500 Chemin de Polytechnique, Montréal, QC H3T 1J4, Canada

**Keywords:** hypoxia, microfluidics, spheroids, tumor microenvironment, CAIX, HIF1-α, radiotherapy, Tirapazamine, sarcoma

## Abstract

**Simple Summary:**

Hypoxia is found in half of the solid cancers and is a major contributor to treatment resistance and promotion of metastasis, leading to shortened patient survival. No user-friendly in vitro preclinical tool exists to study natural chronic hypoxia. The aim of this study was to design a microfluidic device allowing easy culture, maintenance, treatment, and analysis of naturally hypoxic sarcoma spheroids. We confirmed that our jumbo spheroids (>750 µm) contained hypoxic cores, as they expressed the hypoxic marker protein Carbonic Anhydrase IX (CAIX). Quantification of DNA strand breaks from radiotherapy and a hypoxia pro-drug demonstrated hypoxia-dependent treatment responses. Our novel microfluidic device is versatile and convenient for both fundamental and preclinical research, to better understand and treat hypoxic tumors.

**Abstract:**

Hypoxia is a key characteristic of the tumor microenvironment, too rarely considered during drug development due to the lack of a user-friendly method to culture naturally hypoxic 3D tumor models. In this study, we used soft lithography to engineer a microfluidic platform allowing the culture of up to 240 naturally hypoxic tumor spheroids within an 80 mm by 82.5 mm chip. These jumbo spheroids on a chip are the largest to date (>750 µm), and express gold-standard hypoxic protein CAIX at their core only, a feature absent from smaller spheroids of the same cell lines. Using histopathology, we investigated response to combined radiotherapy (RT) and hypoxic prodrug Tirapazamine (TPZ) on our jumbo spheroids produced using two sarcoma cell lines (STS117 and SK-LMS-1). Our results demonstrate that TPZ preferentially targets the hypoxic core (STS117: *p* = 0.0009; SK-LMS-1: *p* = 0.0038), but the spheroids’ hypoxic core harbored as much DNA damage 24 h after irradiation as normoxic spheroid cells. These results validate our microfluidic device and jumbo spheroids as potent fundamental and pre-clinical tools for the study of hypoxia and its effects on treatment response.

## 1. Introduction

Hypoxia is found in 50% to 60% of solid cancers and accounts for 19% to 70% of tumor volume [1,2]. Since the 1950s, hypoxia has been identified as a major contributor to treatment resistance, associated with poor prognosis, metastasis progression and tumor aggressiveness [3,4,5]. For example, soft tissue sarcomas (STSs) are prone to forming large and highly hypoxic tumors, leading to increased risk of metastasis [6,7]. Therefore, investigating combination therapies to target multiple molecular pathways involved in metastasis, such as hypoxia, could potentially improve patient prognosis [8]. The study of hypoxia and its implications on radioresistance have been a topic of interest in fundamental, pre-clinical and clinical research, culminating in 2019 with the Nobel Prize in Physiology or Medicine [9].

**Figure 1:**
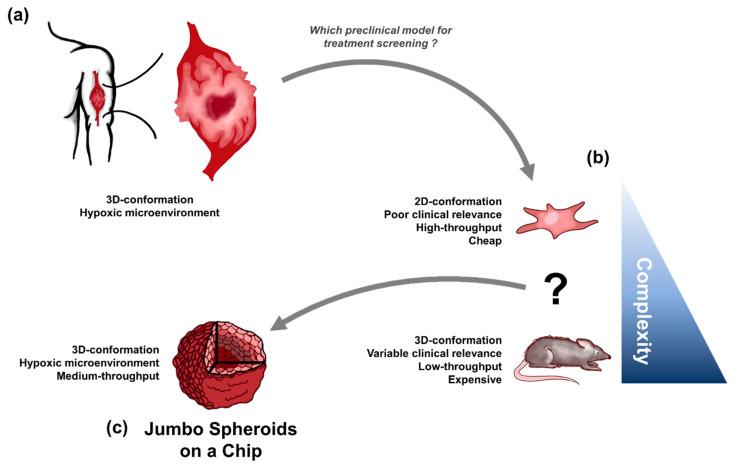
Paradigm for HOnAChip. (**a**) Solid tumors are complex naturally hypoxic 3D-structures. (**b**) Preclinical models of solid tumors fail to easily mimic-clinically relevant characteristics of solid tumors. (**c**) HOnAChip jumbo spheroids are naturally hypoxic tumor spheroids, easily produced in a microfluidic device compatible with drug testing, radiotherapy, and bioanalysis.

In solid tumors, hypoxia is characterized by a lower oxygen concentration than physoxia and appears around 100 µm to 200 µm away from a blood vessel, due to poor and abnormal vasculature [10,11] (Figure 1). Hypoxia is also characterized by the expression of specific genes and proteins belonging to the Hypoxia Responsive Element (HRE) pathway [12,13,14] such as Hypoxia Inducible Factor 1 alpha (HIF1-α) protein and one of its downstream target, Carbonic Anhydrase IX (CAIX), [1,13,15]. Multiple studies demonstrated a correlation between the expression of hypoxia markers such as CAIX and poor prognosis in soft-tissue sarcomas [16,17,18,19].

The efficacy of radiotherapy (RT) is directly linked to hypoxia as oxygen is required to permanently fix DNA damages caused by RT-induced radical species [1,20]. This leads to an oxygen enhancement ratio (OER) of 2–3:1, meaning that there are 2 to 3 times less radiobiological effects in hypoxic cells than in normoxic ones [21]. Moreover, chemoresistance arises from the inability of the abnormal vasculature to properly deliver therapeutic agents to the hypoxic core [1,13]. Finally, hypoxia fosters immunoresistance as immune cells cannot survive in the highly acidic hypoxic microenvironment [22,23,24,25].

Tirapazamine (TPZ, SR-4233), a non-toxic prodrug, was discovered and developed by Brown and Lee in 1986 as an alternative chemotherapy and is one of the first compounds to specifically target hypoxic cells [26]. Activation of TPZ, enabled by local absence of oxygen, results in the production of free radical species and subsequent topoisomerase II-mediated induction of double-strand DNA breaks [26,27]. By acting as a complementary cytotoxin and selectively killing hypoxic cells, the most radioresistant cells in tumors, TPZ enhances the antitumor effects of radiation [1]. Nevertheless, TPZ, as with other hypoxic prodrugs or HIF-targeting agents, failed to translate to clinical improvement [28]. Few drugs in development take hypoxia into account, in part due to the lack of solid user-friendly translational models on which one can study hypoxia and its effects on drugs and RT [1,29,30]. Therefore, there is an unmet need to study the biology of hypoxia at a fundamental and translational level in order to develop better treatment modalities [31].

As of today, no preclinical in vitro tool exists to study hypoxia without artificially inducing it, either with a hypoxic chamber, low O_2_ incubator, chemical such as cobalt chloride (CoCl_2_) or other HIF-1 alpha inhibitors [10,31,32,33]. Artificially inducing hypoxia can present many challenges regarding cell culture and maintaining hypoxia, mainly during treatment or bioanalysis [10,31,33]. Furthermore, artificially hypoxic small spheroids do not recapitulate the oxygen gradients found in normal tumors [31].

Microfluidic technologies have enabled the formation of size-controlled 3D cell cultures with medium to high throughput while being less expensive, more reliable, user-friendly, and faster than other 3D cell culture methods or in vivo techniques [34,35,36,37]. Indeed, animal models are expensive and time-consuming, making them impractical (and ethically questionable) for the first steps of fundamental knowledge finding and pre-clinical studies (Figure 1). Microfluidic 3D tumor models, also called tumor spheroids, can be treated with RT, chemotherapy or drug combinations directly on a chip while being more relevant than 2D cell culture [31,34,35,36,38,39].

It has been reported that spheroids must theoretically have a radius of at least 100 µm to 200 µm, depending on the cell type, to be able to naturally produce gradients of oxygen concentration [10]. However, spheroids must exceed this theoretical threshold to recreate clinically relevant aspects of tumor biology, mainly the coexistence of hypoxic and normoxic cells population in the same structure [31,40]. Few microfluidic devices allow the culture of spheroids large enough (>500 µm of diameter) to adequately mimic tumor geometry, naturally express hypoxia and be useful for bioanalytical and pharmaceutical purposes [10,31,41]. Indeed, spheroid size and density, growth conditions and analytical endpoints have to be taken into careful consideration during the design of a microfluidic chip [31,40,42].

In this study we introduce a simple microfluidic tool capable of reliably producing up to 240 tumor spheroids of >750 µm diameter, the largest spheroids produced on chip so far. We demonstrated how naturally hypoxic spheroids can be used to assess the response to oxygen-dependent treatments TPZ and RT. Results are supported by in silico modeling of oxygen transport and consumption, spatial distribution of gold-standard hypoxic protein, and treatment response measured by immunostaining down to the core of the spheroids.

## 2. Materials and Methods

### 2.1. Microfluidic Chip

#### 2.1.1. Microfluidic Device Fabrication

The top and bottom layers of the chip were cast in polymethyl siloxane (PDMS) Dow SYLGARD 184 Silicone Elastomer Clear (Ellsworth Adhesive, Stoney Creek, ON, Canada) with a 1:10 ratio of Dow SYLGARD 184 curing agent (Dow Corning, Midland, MI, USA). The PDMS-filled molds were placed in a desiccator for 20 min to remove unwanted air bubbles, and then cured at 80 °C for 45 min in a Precision Compact oven (Thermo Fisher Scientific, Saint-Laurent, QC, Canada). Top and bottom layers were unmolded and assembled manually after a 30 s exposure to atmospheric plasma using Enercon plasma gun (Enercon Industries Corporation, Menomonee Falls, WI, USA). Molds fabrication is presented in Appendix A.1.

#### 2.1.2. Microfluidic Device Preparation for Cell Culture

Once the chips were assembled, they were individually put inside an autoclavable box before being autoclaved. Afterwards, chip channels were successively washed once with isopropanol to remove air bubbles, three times with sterilized deionized water and three times with PEG-PPG-PEG, Pluronic^®^ F-108 (Sigma-Aldrich Canada Co, Oakville, ON, Canada) to prevent attachment of biological material. Prepared devices were incubated for 24 h at 37 °C in 5% CO_2_ incubator. Then, chip channels were washed three times with sterilized deionized water and three times with appropriate supplemented culture media before seeding.

### 2.2. Cell Culture

#### 2.2.1. Cell Culture

SK-LMS-1 Human leiomyosarcoma cell line was procured from ATCC (HTB-88, ATCC, Manassas, VA, USA). STS117 Human soft-tissue-sarcoma (STS) primary cell line harboring a loss of function mutation TP53 was derived from patients’ primary extremity STS diagnosed as an undifferentiated pleomorphic sarcoma. STS117 cell line was kindly provided by Dr. R. Gladdy (Mount Sinai Hospital, Toronto, ON, Canada) [17]. SK-LMS-1 was cultured in EMEM (Wisent Inc., St-Bruno, QC, Canada), STS117 was cultured in DMEM F12 (Wisent Inc.), both supplemented with 10% Fetal Bovine Serum (FBS) (Gibco, Thermo Fisher Scientific, Saint-Laurent, QC, Canada) and 1% Penicillin–Streptomycin solution (Wisent Inc.). SK-LMS-1 and STS117 cells were maintained by subculturing at 80% confluency. Briefly, the medium was aspirated, cells were washed with Phosphate Buffer Saline (PBS) (Wisent Inc.) and were trypsinized with 0.025% trypsin EDTA (Wisent Inc.) for 2–3 min at 37 °C. Once cells are detached, supplemented medium was added to stop the enzymatic reaction and the cell suspension was centrifuged for 5 min at 1500 rpm. The cell pellets were resuspended in the appropriate volume to perform seeding in the microfluidic devices.

#### 2.2.2. Spheroid Formation

Upon preparation of the microfluidic device, suspended cells were seeded at a concentration of 3 × 10^−6^ cells/mL. An amount of 200 µL of cell suspension was pipetted in the device thrice in the inlet and thrice in the outlet for homogenization. The medium was then changed every 24 h until spheroid were formed 2 days after seeding. Control spheroids formation and hypoxia induction for control spheroids methods are presented in Appendix A.2.

### 2.3. Hypoxic Protein Analysis

#### 2.3.1. Western Blot

The top layer of the device was peeled off manually to allow spheroids retrieval. Fifteen spheroids were pipetted into Eppendorf tubes, washed twice with PBS and centrifugated at 2 min^−1^·g for 2 min. PBS was then removed, and samples were put on ice. Spheroids were then homogenized 3 times 3 s using a sonicator (XL-2000, Misonix, Cole-Parmer Canada Company, Montréal, QC, Canada) in RIPA lysis buffer (Sigma-Aldrich, Louis, MO, USA) supplemented with Phosphatase Inhibitor PhosSTOP (1:1000) (Roche, Sigma-Aldrich) and cOmplete™ Protease Inhibitor Cocktail (1:10,000) (Roche, Sigma-Aldrich) to prevent denaturation. Then samples were centrifugated for 15 min at 13,000 rpm at 4 °C (Biofuge pico, Heraeus, Kendro Laboratory Products, Asheville, NC, USA). Protein concentration was determined by BCA Protein Assay (Bio-Rad Laboratories Ltd., Saint-Laurent, QC, Canada and lysates concentrations were standardized to 1 µg/µL by diluting in 4× Laemmli buffer (Bio-Rad, Hercules, CA, USA) and 100 mM DTT. Before blotting, samples were heated at 100 °C for 7 min. Then, 30 g of cell lysates were loaded in precast gels 4–15% Mini-PROTEAN^®^ TGX™ (Bio-Rad) and transferred onto PVDF membrane (Immobilon-P, Merck Millipore, Sigma-Aldrich). PBST (PBS 1×, 0.1% Tween-20) and 5% skim milk solution was used for blocking. Membranes were then incubated, at 4 °C overnight, with primary antibody diluted in blocking buffer; 1:750 rabbit anti-HIF1-α (ab179483, Abcam, Waltham, MA, USA), 1:2000 rabbit anti-CAIX (ab15086, Abcam, USA) and 1:20,000 mouse anti β-actin (A-5441, Sigma-Aldrich). After washing with PBST buffer, membranes were incubated 1 h at room temperature in HRP (CellSignaling, Withby, ON, Canada) diluted in blocking buffer. Then, membranes were rinsed, incubated for 1 min with SuperSignal™ West Pico PLUS Chemiluminescent Substrate (ThermoFisher Scientific, Waltham, MA, USA) and imaged on Bio-Rad ChemiDoc MP Imaging system (Bio-Rad). Chemiluminescent intensities were measured and HIF1-α and CAIX levels were normalized by β-actin levels to eliminate influence of loading differences. The ratios were then normalized to the positive control.

#### 2.3.2. Immunofluorescence

Two days after seeding or 24 h after treatment, spheroids were washed 3 times with PBS directly in the device and fixed with Formalin 10% (Fisher Scientific Company, Toronto, ON, Canada) for 45 min. Then, spheroids were washed 5 times with PBS, the top layer of the chip was peeled off to proceed to inclusion of 5 spheroids in optical cutting compound OCT (Leica, Buffalo Grove, IL, USA). Included samples were left overnight to sediment, thereby insuring homogeneous z-levels across samples. Afterwards, included samples are frozen on dry ice and stored at −80 °C. Frozen samples were sectioned using Leica Cryostat (Leica, Buffalo Grove, IL, USA) with a 5 µm thickness at −20 °C. Sections were incubated for 1 h at room temperature with blocking buffer (PBS 1×, 3% IgG-free, Protease-free BSA, 0.5% Triton 100 10×). Sections were then incubated in blocking buffer with rabbit anti-CAIX (1:1000) (PA1-16592, ThermoFisher Scientific, Waltham, MA, USA) and mouse anti-γH2AX (1:250) (05-636, EMP Millipore) overnight at 4 °C. After washing 3 times with PBS, sections were incubated in secondary antibody buffer (PBS 1×, 3% BSA) with AlexaFluor-647 antibody (1:750) (A31573, Invitrogen, USA) and AlexaFluor-488 antibody (1:750) (A31571, Invitrogen, ThermoFisher Scientific, Waltham, MA, USA) for 1 h at room temperature. Afterwards, sections were stained with DAPI (1:5000 from 5 mg/mL stock solution) (D3571, Invitrogen, USA) to stain nuclei. Then, sections were mounted with ProLong™ Gold Antifade Mountant (P36934, Invitrogen, ThermoFisher Scientific, Waltham, MA, USA). Images were obtained on a Zeiss fluorescence microscope with Axio-Vision 4.0 software (Carl Zeiss AG, Jena, Germany). Images were analyzed using ImageJ (ImageJ, Fiji General Public License). Image analysis is described in Appendix A.3.

#### 2.3.3. In Silico Modeling of Oxygen Consumption

A finite element method with the commercial COMSOL Multiphysics^®^ software was used to model the oxygen consumption in our jumbo and small spheroids. A 2D-symmetry approximation was used to draw the spheroids as perfect circles, with a previously estimated mean diameter. Oxygen consumption was defined by a reaction equation with Michaelis–Menten kinetics, using a method derived from Grimes et al. [43]. Briefly, Michaelis–Menten parameters were set for our jumbo spheroids so that CAIX expression threshold of 10 mmHg (0.0126 mM) was crossed at the estimated mean depth of hypoxic region. After adjusting the oxygen consumption rates for size, the model was then applied to small spheroids. Michaelis–Menten constant was set at 4.63 × 10^−3^ mol/m^3^ [34]. Oxygen consumption rates applied to the models can be found in Table 1.

### 2.4. Treatment Modalities

#### 2.4.1. Tirapazamine (TPZ)

Serial dilution of Tirapazamine (SML0552, Sigma; dissolved in DMSO at 50 mM) in proper culture media was performed to reach desired concentration of 10 µM and 35 µM (a dose within the range used in human trial [28]) (control: culture media, 1:1500 DMSO). Treatment with TPZ was performed 2 days after seeding by pipetting 5 × 200 µL in each channel to ensure homogeneity. Then, devices were incubated for 24 h at 37 °C in 5% CO_2_ incubator.

#### 2.4.2. Conventional Radiotherapy

Two days after seeding, fresh media were added to each channel before irradiation of spheroids directly on chip using the Gammacell 3000 irradiator (Best Theratronics, Ottawa, ON, Canada) at defined doses (0, 2, 4 and 8 Gy).

#### 2.4.3. Combination Therapy

Combination of TPZ and radiation therapy was performed directly on a chip. Spheroids were irradiated 2 days after seeding at 0, 2, 4 or 8 Gy, then treated with either 0, 10 or 35 µM of TPZ and incubated for 24 h at 37 °C in 5% CO_2_ incubator. Then, spheroids were retrieved 24 h after treatment and analyzed by IF assay as previously described.

### 2.5. Statistical Analysis

Statistical analysis was conducted using GraphPad Prism (Version 9.0.1, GraphPad, San Diego, CA, USA). Gaussian distribution of SK-LMS-1 and STS117 jumbo spheroids diameter were assessed by performing a Shapiro-Wilk lognormality and normality test. Distribution of samples passed the normality test if *p* > 0.05. For Western blot analysis mean fold changes in proteins in negative control (small-normoxic), positive control (small-hypoxic) and jumbo spheroids were analyzed for significance using an ordinary one-way ANOVA with Tukey’s multiple comparisons test. For IF analysis, comparison of γH2AX foci per nuclei area between normoxic and hypoxic region of jumbo spheroids cross-section, when treated with either TPZ or RT alone, were analyzed for significance using an ordinary one-way ANOVA with Šídák’s multiple comparisons test. Comparison of γH2AX foci per nuclei area in normoxic and hypoxic region of jumbo spheroids cross-section, when treated with a combination of TPZ and RT, were analyzed for significance using an ordinary one-way ANOVA with Tukey’s multiple comparisons test.

## 3. Results

### 3.1. The Microfluidic Chip Allows Formation of Size-Controlled Jumbo Spheroids

**Figure 2:**
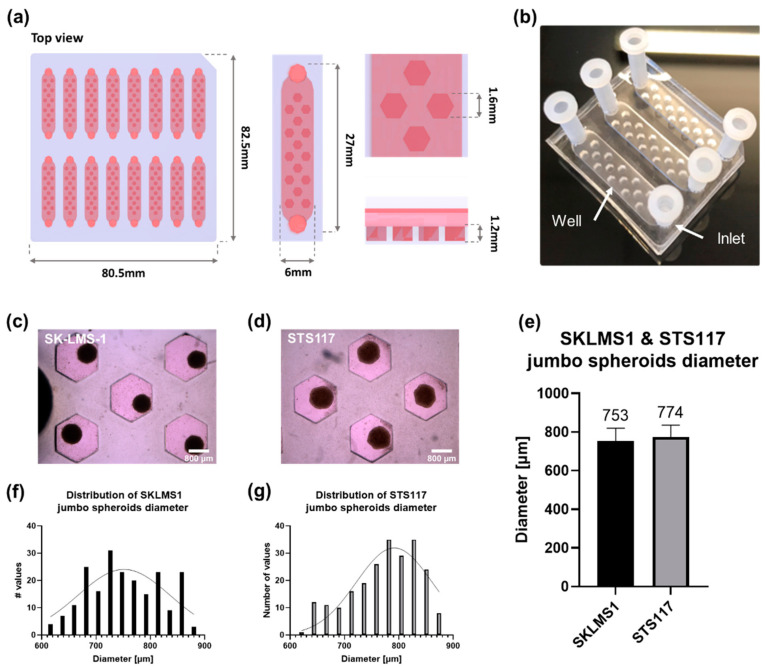
Spheroids of 2 sarcoma cell lines are successfully formed 2 days after seeding and present a controlled diameter. (**a**) 3D-render of assembled chip. (**b**) Image of assembled chip (3 channels). Plastic inlets are separate from the chip and added for experimental purposes. (**c**,**d**) Brightfield image of SK-LMS-1 (**c**) and STS117 (**d**) jumbo spheroids 2 days after seeding. (**e**). Mean diameter of SK-LMS-1 and STS117 jumbo spheroids 2 days after seeding. Diameter of SK-LMS-1 spheroids is 753 ± 67 µm, *n* = 210. Diameter of STS117 spheroids is 774 ± 62 µm, *n* = 227. Values are presented as mean ± SD. Results were obtained across more than 3 repetitions. (**f**,**g**) Distribution of SK-LMS-1 (**f**) and STS117 (**g**) jumbo spheroids diameter. In both cell lines, spheroids diameters follow a Gaussian distribution (SK-LMS-1: *p* = 0.53, STS117: *p* = 0.69).

The microfluidic chip consists of 16 independent channels, each containing 15 wells allowing formation of one jumbo spheroid each (Figure 2a). Hexagonal-shape wells were chosen to optimize the number and volume of wells per channel (Figure 2a). Final dimensions for the wells are 1.6 mm for the inscribed diameter and a depth of 1.2 mm. The chip was optimized to be the same dimension as a 96-well plate, as well as being compatible with an 8-channels multipipette and for each channel to fit in a histology cassette. In comparison with a 96-well plate, this microfluidic chip allows the formation of 240 jumbo spheroids, i.e., 3 times more spheroids per mm^2^, while using 2 times less reagent than in standard protocols [44]. In addition, this chip is optically translucent, allowing imaging of spheroids with brightfield microscopy, and can be cut with a scalpel or a razor blade for experimental purposes (Figure 2b). Sterilization of the chip can be performed using a standard autoclave and the chips can be kept for at least one year after fabrication without alteration of their physicochemical properties.

In both cell lines, spheroids are successfully formed 2 days after seeding and present a homogeneous diameter following a gaussian distribution (Figure 2c,d,f,g). SK-LMS-1 jumbo spheroids have a diameter of 753 ± 67 µm and STS117 jumbo spheroids have a diameter of 774 ± 62 µm (Figure 2e).

### 3.2. Presence of Hypoxia in Small vs. Jumbo Spheroids

**Figure 3:**
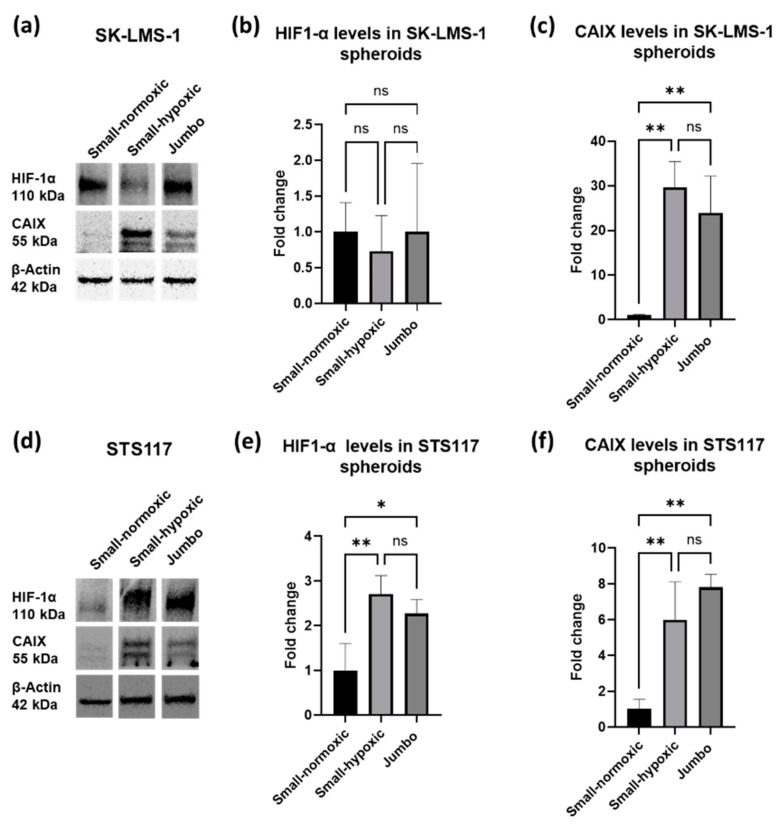
In both cell lines, CAIX expression increases significantly in jumbo spheroids, consistent with presence of hypoxia. (**a**,**d**) CAIX and HIF-1α expression in SK-LMS-1 (**a**) and STS117 (**d**) small-normoxic (diameter < 450 µm), small-hypoxic and jumbo (diameter > 750 µm) spheroids. (**b**,**e**) Fold change in HIF1-α protein in SK-LMS-1 (**b**) and STS117 (**e**) small-normoxic, small-hypoxic and jumbo spheroids (STS117: small-normoxic vs. small-hypoxic: *p* = 0.0089; small-normoxic vs. jumbo: *p* = 0.0320). (**c**,**f**) Fold change in CAIX protein in SK-LMS-1 (**c**) and STS117 (**f**) small-normoxic, small-hypoxic and jumbo spheroids (SK-LMS-1: small-normoxic vs. small-hypoxic: *p* = 0.0022; small-normoxic vs. jumbo: *p* = 0.0069), (STS117: small-normoxic vs. small-hypoxic: *p* = 0.0087; small-normoxic vs. jumbo: *p* = 0.0018). Values are presented as mean ± SD (standard deviation), *N* = 3 with more than 15 spheroids per repetition, * *p* < 0.05, ** *p* < 0.005, ns: non significant.

A high expression of HIF1-α was observed in Western blots of SK-LMS-1 spheroids, regardless of their size (Figure 3a,b and Figure S1). Although there is a high expression of HIF1-α protein in SK-LMS-1 jumbo spheroids, no statistical difference between small-normoxic spheroids (negative control, <450 µm), small-hypoxic spheroids incubated for 24 h in 2% O_2_ incubator (positive control, method in Appendix A.2) and jumbo spheroids was observed (Figure 3b). In contrast, Western blot analysis for STS117 showed the expected variation in protein levels (Figure 3d,e and Figure S1). STS117 small-hypoxic and jumbo spheroids expressed 2.7 times and 2.3 times more HIF-α, respectively, than small-normoxic ones (Figure 3e). No statistical difference was observed between STS117 jumbo spheroids and small-hypoxic ones.

CAIX expression levels were consistent with our expectations in both cell-lines, namely a low expression of CAIX in small-normoxic spheroids and a high expression of CAIX in small-hypoxic and jumbo spheroids (Figure 3a–c,f). Indeed, SK-LMS-1 small-hypoxic and jumbo spheroids expressed 30 times and 24 times more CAIX, respectively, than small-normoxic spheroids (Figure 3c). No statistical difference was observed between SK-LMS-1 jumbo spheroids and positive control. Similarly, in STS117, small-hypoxic and jumbo spheroids expressed 6 times and 7.8 times more CAIX, respectively, than small-normoxic spheroids (Figure 3f).

**Figure 4:**
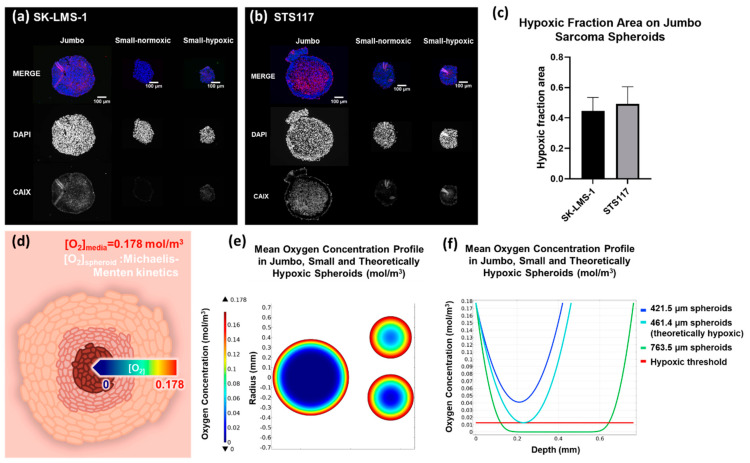
Hypoxia is localized in the core of jumbo spheroids at expected depths. (**a**,**b**) CAIX (red) staining in SK-LMS-1 (**a**) and STS117 (**b**) small (normoxic and hypoxic controls) and jumbo spheroids using anti-CAIX antibody. *N* = 3 with more than 3 spheroids per repetition. Scale bar = 100µm. (**c**) Hypoxic fraction area on jumbo sarcoma spheroids. 45% ± 8.9% of SK-LMS-1 and 49% ± 11.4% of STS117 cross section area express CAIX and is defined as hypoxic area. Results are presented as mean ± SD. *N* = 3. (**d**) Schematic of oxygen consumption modelling in jumbo spheroids. Michaelis-Menten parameters for oxygen consumption are derived from CAIX staining. (**e**) Mean oxygen concentration profile in jumbo and small spheroids cross-section. (**f**) Graph of mean oxygen concentration in small, jumbo and theoretically hypoxic spheroids cross-section.

An immunofluorescence assay (IF) was performed to localize the expression of CAIX in small-normoxic, small-hypoxic, and jumbo spheroids cross-section. As expected, in both SK-LMS-1 and STS117, CAIX is localized in the core of the jumbo spheroids (Figure 4a,b). Respectively, 45% of SK-LMS-1 jumbo spheroids cross-section area and 49% of STS117 jumbo spheroids cross section area are CAIX-positive (Figure 4c).

**Figure A1:**
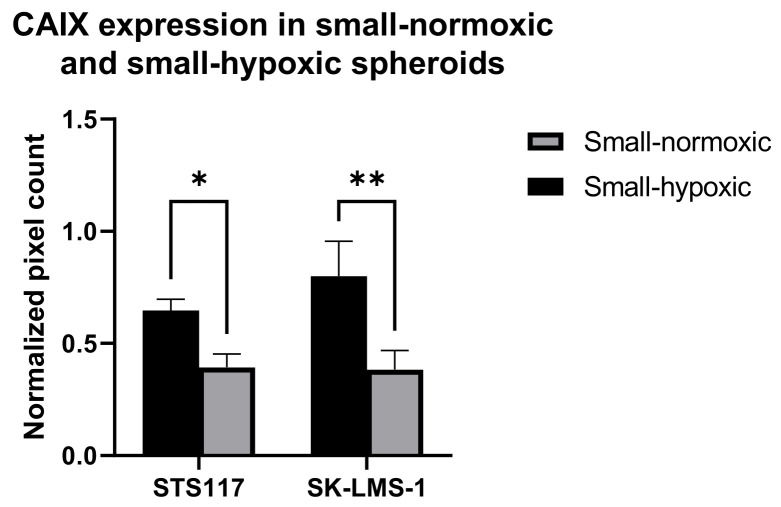
CAIX expression is significantly higher in small-hypoxic spheroids than in small-normoxic ones. Normalized pixel count of CAIX IF staining, in STS117 and SK-LMS-1 small normoxic and small hypoxic spheroids (STS117: small-normoxic vs. small hypoxic: *p* = 0.0254. SK-LMS-1: small-normoxic vs. small hypoxic: *p* = 0.0015). Values are presented as mean ± SD, *N* = 3, 2 spheroids per repetition, * *p* < 0.05, ** *p* < 0.005.

For both cell lines, small-normoxic spheroids expressed significantly less CAIX signal than small-hypoxic ones (Figure A1) CAIX was expressed all over the section of small-hypoxic spheroids, as expected for hypoxia-induced spheroids (Figure 4a,b).

Using the IF results, we calculated the mean depth of hypoxic regions in our jumbo spheroids as:

$$d_{h}=r\times \left(1-\sqrt{\frac{A_{h}}{A}}\right)$$

With $d_{h}$ the mean hypoxic depth, r the mean total radius, and $\frac{A_{h}}{A}$ the hypoxic fraction area in our jumbo spheroids with *A_h_* the CAIX-positive area and *A* the total area of the spheroid (based on the CAIX expression in the IF). This gave us an estimated hypoxic depth of 120 µm in jumbo spheroids. Given these values, we built a numerical model of oxygen consumption in both our jumbo and small spheroids to understand why small spheroids (<450 µm) did not express CAIX (Figure 4d). As shown in Figure 4e,f, in a model specifically built so that CAIX-expression threshold is crossed at the previously calculated depth, the same threshold is never crossed in smaller spheroids. Using our model, we estimated that STSs spheroids should theoretically be at least 461.4 µm wide for the first cell to cross the hypoxic threshold, confirming the necessity of spheroids larger than 750 µm to have a meaningful number of hypoxic cells.

### 3.3. Evaluation of Treatment Response in Jumbo Spheroids

SK-LMS-1 and STS117 spheroids were treated with RT (0, 2, 4, 8 Gy) and TPZ (0, 10, 35 µM; a dose within the range used in human trial [28]), both of which inflict DNA damages as their main mode of action [13]. As previously stated, TPZ preferentially causes single- and double-DNA strand breaks in hypoxic cells [45]. Therefore, cytotoxicity of TPZ in both SK-LMS-1 and STS117 jumbo spheroids was assessed by quantifying double strand DNA breaks using γH2AX marker by IF.

First, we compared the number of γH2AX foci per nuclei area in the CAIX-positive region (hypoxic region) versus CAIX-negative region (normoxic region) of the jumbo spheroids cross-section, in both cell lines, when treated with either TPZ or RT only.

**Figure 5:**
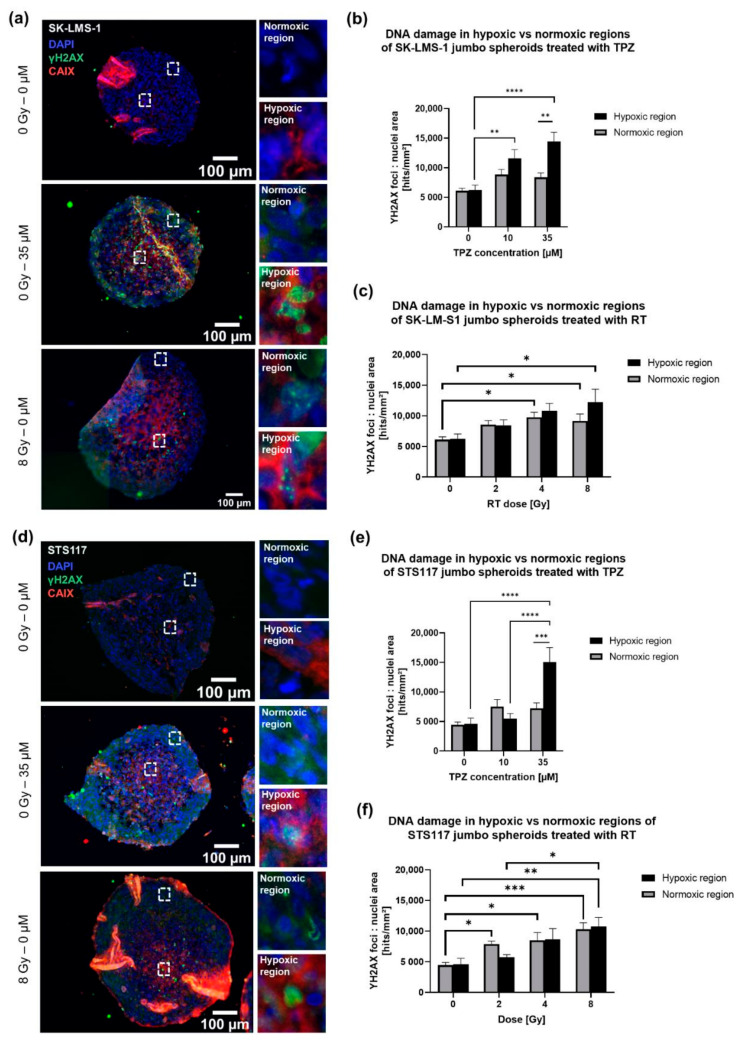
Contrary to RT alone, treatment with TPZ alone resulted in oxygen-dependent responses. (**a**,**d**) DNA damages (γH2AX, green), hypoxia (CAIX, red) and nuclei (DAPI, blue) staining in SK-LMS-1 (**a**) and STS117 (**d**) jumbo spheroids treated with either 35 UM of TPZ or 8 Gy of RT. (**b**,**c**,**e**,**f**) DNA damages in hypoxic (H) versus normoxic (*N*) regions of SK-LMS-1 and STS117 jumbo spheroids treated with either TPZ alone (**b**,**e**) or with RT alone (**c**,**f**) (SK-LMS-1: H: 0–10 µM: *p* = 0.0038, 0–35 µM: *p* < 0.0001; 35 µM: H vs. *N*: *p* = 0.0096), (STS117: H: 0–35 µM: *p* < 0.0001, 10–35 µM: *p* < 0.0001; 35 µM: H vs. *N*: *p* = 0.0009), (SK-LMS-1: H: 0–8 Gy: *p* = 0.0157; *N*: 0–4 Gy: *p* = 0.0111, 0–8 Gy: *p* = 0.043), (STS117: H: 0–8 Gy: *p* = 0.0076, 2–8 Gy: *p* = 0.0429; *N*: 0–2 Gy *p* = 0.0387, 0–4 Gy: *p* = 0.0125, 0–8 Gy: *p* = 0.0002). From γH2AX IF staining foci were counted and normalized per nuclei area. Values are presented as mean ± standard error of the mean (SEM), *N* = 3–4, 2–4 spheroids per repetition, * *p* < 0.05, ** *p* < 0.005, *** *p* < 0.0005, **** *p* < 0.0001.

Both SK-LMS-1 and STS117 hypoxic regions show a dose–response relationship to TPZ (Figure 5a,d). Indeed, there is a dose-dependent response in hypoxic regions only, with significant differences between 0 µM and 10 µM for SK-LMS-1, between 10 µM and 35 µM for STS117, and 0 µM and 35 µM for both cell lines (Figure 5b,e). Moreover, at 35 µM there is a significant difference between the hypoxic regions and the normoxic regions, confirming that TPZ induces DNA damage preferentially to hypoxic regions as previously demonstrated in the literature (Figure 5b,e).

However, no statistical differences were observed between normoxic and hypoxic regions of STS117 and SK-LMS-1 jumbo spheroids when treated with RT only (Figure 5c,f). The increase in γH2AX foci per nuclei area is dose-dependent in both cell lines regardless of the region. In the absence of TPZ, significant differences are observed in hypoxic regions between 0 Gy and 8 Gy for both cell lines, and 2 Gy and 8 Gy for STS117 (Figure 5c,f). These differences are already observed between 0 Gy and 4 Gy, and 0 Gy and 8 Gy in SK-LMS-1 normoxic regions, and between 0 Gy and 2 Gy, 0 Gy and 4 Gy and, 0 Gy and 8 Gy in STS117 normoxic regions (Figure 5c,f). Despite no significant differences between regions for a given radiation dose, these results could indicate that a higher radiation dose is required to yield significant DNA damages in hypoxic regions than in normoxic regions.

**Figure 6:**
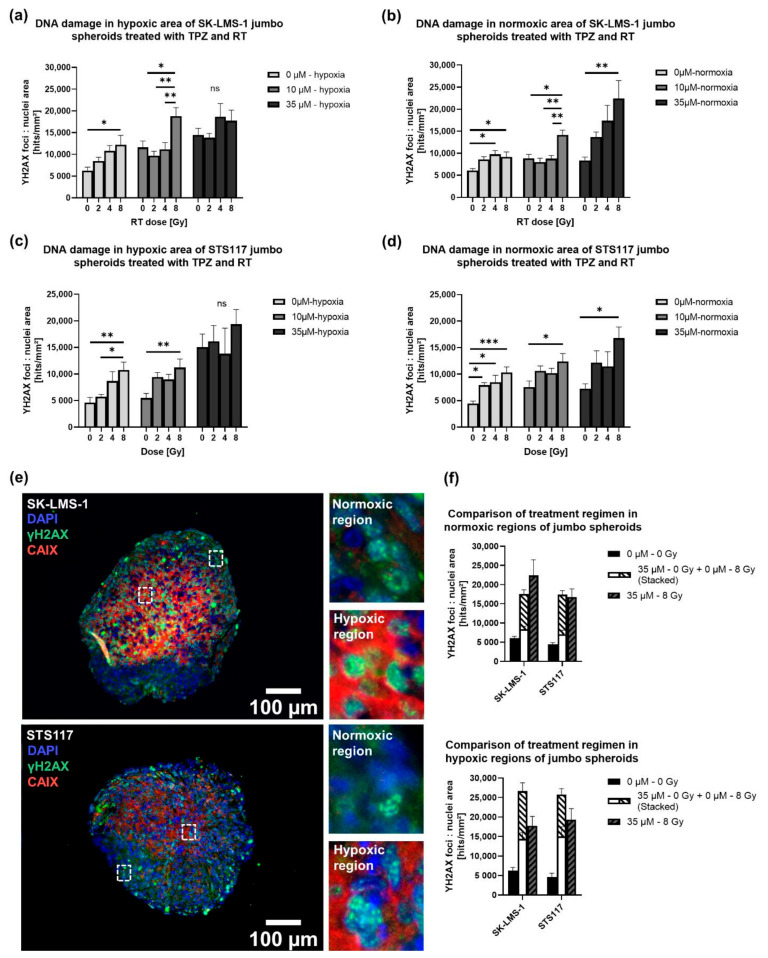
Combination of RT and TPZ resulted in oxygen-dependent responses. (**a**–**d**) DNA damages in hypoxic area (**a**), (**c**) and normoxic area (**b**), (**d**) of SK-LMS-1 and STS117 jumbo spheroids treated with TPZ and RT, (SK-LMS-1: H: 10 µM: 0–8 Gy: *p* = 0.018, 2–8 Gy: *p* = 0.0023, 4–8 Gy: *p* = 0.0076; *N*: 10 µM: 0–8 Gy: *p* = 0.0015, 2–8 Gy: *p* = 0.0003, 4–8 Gy: *p* = 0.0008; *N*: 35 µM: 0–8 Gy: *p* = 0.0077), (STS117: H: 10 µM: 0–8 Gy: *p* = 0.0072; *N*: 10 µM: 0–8 Gy: *p* = 0.0294; *N*: 35 µM: 0–8 Gy: *p* = 0.0126). (**e**) DNA damages (γH2AX, green), hypoxia (CAIX, red) and nuclei (DAPI, blue) staining in SK-LMS-1 and STS117 jumbo spheroids treated with 35 µM of TPZ and 8 Gy of RT. (**f**) Comparison of treatment regimen in normoxic and hypoxic regions of jumbo spheroids. From γH2AX IF staining, foci were counted and normalized per nuclei area. Values are presented as mean ± standard error of the mean (SEM), *N* = 3–4, 2–4 spheroids per repetition, * *p* < 0.05, ** *p* < 0.005, *** *p* < 0.0005.

Then, we investigated the combined effects of TPZ and RT and compared the DNA damages in each region separately. For SK-LMS-1, at 10 µM of TPZ, significant differences are observed between 0 Gy and 8 Gy, 2 Gy and 8 Gy, and 4 Gy and 8 Gy in both hypoxic and normoxic regions (Figure 6a,b). For STS117, at 10 µM of TPZ, significant differences are observed only between 0 Gy and 8 Gy in both hypoxic and normoxic regions (Figure 6c,d).

In the normoxic regions of both cell lines, at 35 µM of TPZ, RT retains its observed dose-dependent DNA damage increase (Figure 6b,d). Furthermore, for SK-LMS-1 and STS117, combination therapy of 35 µM and 8 Gy appears to yield similar damages than the sum of each condition alone (Figure 6e,f). In the hypoxic regions of both cell lines, there are no significant differences between each RT dose (either 0 Gy, 2 Gy, 4 Gy and 8 Gy) when jumbo spheroids are treated with 35 µM of TPZ (Figure 6a,c). Although the difference is not significant, combination therapy of 35 µM and 8 Gy appears to yield fewer damages than the sum of each condition alone (Figure 6e,f). These results could indicate additive effects of the combination therapy under normoxia, in SK-LMS-1 and STS117, respectively.

## 4. Discussion

Our microfluidic chip allows the formation of up to 240 size-controlled jumbo hypoxic spheroids in 48 h with minimal and user-friendly operation, on which rapid evaluation of treatment combination can be performed. Natural expression of hypoxia in our spheroids alleviates the challenges presented by artificial induction. Indeed, to artificially maintain culture media and cells under low pO_2_, nitrogen needs to be injected in the chamber [10,33]. This leads to a progressive acidification of the culture media, which has been shown to alter drug conformation and possibly inactivate them [13,46]. Furthermore, studying RT on hypoxic cell cultures would either require for the whole chamber to fit in the irradiator or for the hypoxic environment to be interrupted for the duration of the treatment. Finally, artificial induction of hypoxia does not allow both normoxic and hypoxic regions within the same sample, thus making it less consistent with real tumor microenvironment. Furthermore, compared to hanging drops or spinner flask methods, our chip enables a precise control of spheroid size while being more ergonomic. Control of spheroids size is achieved by the optimization of cell density that needs to be seeded and by the size of the wells [38]. Compared to a 96-well plate, the increased number of samples per condition reduces the amount of reagent required and the experimental variability. Our device allows easy drug delivery, retrieval of samples for standard histopathology or bioanalysis and is fully compatible with RT.

Protein quantitation demonstrated higher levels of gold-standard hypoxic protein CAIX in our jumbo spheroids, illustrating the presence of chronic natural hypoxia without resorting to hypoxic chamber or chemicals. Quantitation of other gold-standard hypoxic protein HIF1-α yielded mixed results depending on the cell line, varying as expected between samples in STS117 but not in SK-LMS-1. As HIF1-α levels are known to vary between cell lines, our failure to correlate HIF1-α expression and hypoxia in SK-LMS-1 could be attributed to previously observed abnormal HIF1-α basal levels [47,48]. Indeed, SK-LMS-1 exhibits an altered MYC pathway (amplification of *MYCBPAP* and deletion of *MLX* and *MXI1*) which has been shown to drive HIF1-α stabilization under normoxia [21,49,50,51]. As 3D-conformation has been shown to influence gene expression and regulation in tumor cells, investigating *MYC*, *HIF1A*, *VHL* and *PHD* gene expression and relevant miRNAs levels could provide insights into the significance of HIF1-α levels in SK-LMS-1 jumbo spheroids [52]. SK-LMS1 may not be the best sarcoma model, but served to exemplify a model in which a disconnect between HIF1-α and CAIX occurs in the 3D model, which had been observed in vivo [53]. Overall, our results in jumbo spheroids indicate that CAIX is a more reliable marker of hypoxia across cell lines, as previously observed clinically [2,54,55,56].

Histopathology analysis showed that CAIX-expressing cells were localized at the center of our jumbo spheroids, at depths similar to those found in the literature, strengthening our claim of natural expression of hypoxia [12]. CAIX/HIF1-α co-staining would have provided meaningful information on protein localization in our jumbo spheroids, especially for SK-LMS-1 as they expressed high level of HIF1-α even in normoxia, but proved extremely arduous and not cost-effective mainly due to the high HIF1-α antibody concentration required for IF [57]. Indeed, studies have highlighted how hazardous IF staining of HIF1-α is, often preferring CAIX or pimonidazole [8,58,59,60]. The CAIX signal displayed at the rim of our jumbo spheroids is attributed, as other staining artifacts, to local folding of sample, and could be reduced by using positively charged glass slides.

Using an in silico model of oxygen consumption derived from IF results of jumbo spheroids, we were able to show that for STS cell lines, observed expression of hypoxia in jumbo spheroids implied an absence of hypoxia in smaller spheroids. This observation highlights how the link between spheroid cellular density and spheroid volume drives the oxygen consumption, hence the expression of hypoxia [61,62]. Indeed, extremely high cellular density has been shown to upregulate HIF1-α even in 2D cell culture [62]. As the size of jumbo spheroids makes them too small to use microneedle probes, the standard method for pO2 measurements, our model is still limited by the assumption that CAIX begins to be expressed at 10 mmHg [43,58,63,64,65,66].

**Figure A2:**
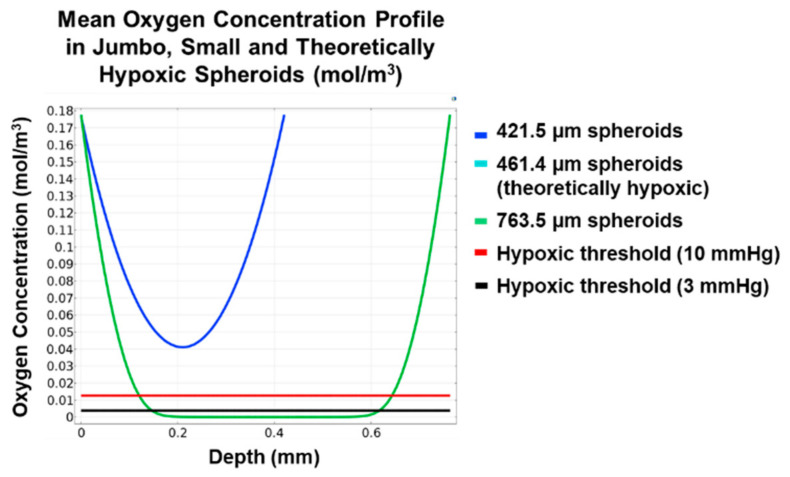
80% of hypoxic area is under OER = 2 threshold (3 mmHg, black line). Graph of mean oxygen concentration in small, jumbo and theoretically hypoxic spheroids cross-section.

We used our microfluidic device to test combined treatment of jumbo spheroids with conventional RT and widely used hypoxia pro-drug TPZ [28,67,68,69]. As expected, TPZ was preferentially cytotoxic to CAIX-expressing cells in a dose-dependent manner, thereby cementing our spheroids as a naturally hypoxic 3D tumor model [27]. Surprisingly, both hypoxic and normoxic regions of our jumbo spheroids displayed a dose-response effect to RT, contradicting with expected hypoxia-associated radioresistance. A possible explanation could be that, although our spheroids are hypoxic enough to express CAIX, oxygen levels are still high enough to prevent radioresistance. Indeed, an OER of 2 has been documented to appear below 3 mmHg, which according to our in silico model would only represent a fraction of our hypoxic core (Figure A2) [70]. Therefore, increasing the size of our jumbo spheroids might increase this fraction enough to observe radioresistance. Another explanation could be that, 24 h after treatment, the oxygen-dependent DNA-repair in normoxic regions and the accumulation of DNA-damages in hypoxic regions are important enough to cancel out differences between these two regions [21,71]. We chose to look at γH2AX staining 24 h after treatment, based on the study of Olive et al. [72], which aids in prediction of tumor response to treatment and gives us a better indication on treatment efficacy. As hypoxic cells are known to have impaired DNA-repair and a tendency to accumulate DNA damages, while DNA of normoxic cells can repair themselves more efficiently, investigating γH2AX foci 30 min after irradiation could reveal a potential hidden radioresistance in our jumbo spheroids [71,72,73].

Combination of RT and TPZ resulted in oxygen-dependent responses. Under hypoxia, RT loses its dose-response effect when combined with 35 µM of TPZ (a dose within the range used in human trial [28]) but not with 10 µM, suggesting that high concentration of TPZ masks additional effects of RT. However, under normoxia, 35 µM of TPZ increased efficiency of 8 Gy of RT additively for STS117 and SK-LMS-1, as previously observed in preclinical studies [74,75].

At the clinical level, additional benefits of TPZ combined with RT and other chemotherapeutic agents such as cisplatin and carboplatin have not been demonstrated which could be explained by poor patient-stratification for hypoxia in most of these studies [28,75]. Our results suggest that repurposing TPZ in combination with RT alone could yield meaningful results in soft tissue sarcoma treatment.

Finally, characterization of jumbo spheroids multilayered structure (presence of senescent, quiescent cell and/or necrotic cells) and expression of Hypoxia Responsive Element genes would provide meaningful information on our hypoxic tumor model, and how it can be used for the study of hypoxia.

## 5. Conclusions

To date, our spheroids are the largest (>750 µm) on a chip and display a hypoxic core expressing gold-standard hypoxia-associated protein CAIX at the expected depth. Remarkably, this feature is absent in smaller spheroids of the same cell line, as shown both experimentally and in silico. The microfluidic format used is simple, reliable, and fully compatible with RT. As a proof of concept, jumbo spheroids were treated with TPZ hypoxic prodrug and RT, the efficacies of which are oxygen-dependent thus linked to hypoxia. In both the SK-LMS-1 and STS117 cell line, treatment with TPZ led to dose-dependent response in their hypoxic core only. Conversely, both hypoxic and normoxic regions of jumbo spheroids displayed a dose-response effect to RT, showing no evidence of radioresistance in the hypoxic core. Interestingly, the combination of both TPZ and RT resulted in an oxygen-dependent response. Overall, addition of 8 Gy of RT to 35 µM of TPZ yielded maximum DNA damages to both normoxic and hypoxic region, in an additive manner.

Finally, using our microfluidic device and our jumbo hypoxic spheroids to explore the biology of hypoxia and its implication on treatment resistance on a variety of cancer types would provide a useful preclinical tool for drug screening and treatment combinations.

## Figures and Tables

**Table 1:** Parameters used in the oxygen consumption in silico model.

Spheroids	$\mathrm{Oxygen}\;\mathrm{Consumption}\;\mathrm{Rate}\;[\mathrm{mol}/m^{3}\cdot s]$
SKLMS1 jumbo	3.65 × 10^−2^
STS117 jumbo	4.07 × 10^−2^
SKLMS1 small	2.61 × 10^−2^
STS117 small	2.33 × 10^−2^

## Data Availability

The data presented in this study are available on request from the corresponding author.

## Supplementary Materials

The following are available online at https://www.mdpi.com/article/10.3390/cancers13164046/s1, Figure S1: Uncropped Western Blot Figures.

## Appendix A

### Appendix A.1. Mold Fabrication

Prototype molds of microfluidic devices were designed with CAD software CATIA (Dassault Systems, Vélizy-Villacoublay, IDF, France) and 3D-printed with GR-1 clear resin (Pro3dure Medical LLC, Eden Praire, MN, USA) using Asiga MAX X27 printer (Asiga, Alexandria, Australia) for fast prototyping. All 3D-printed molds were washed 3 times for 10 min in isopropanol 100% (Fisher Scientific Company, Toronto, ON, Canada) right after printing, and then cured under UV lamp for 5 min. Afterwards, molds were placed in a Precision Compact oven (ThermoFisher Scientific, Waltham, MA, USA) at 80 °C for 2 h in order to remove remaining traces of chemicals. After optimizing the design, final molds were CNC-machined with Modela MDX-40A 3D Milling Machine (Roland, Irvine, CA, USA) and the tool path was designed with Fusion 360 (Autodesk, Mill Valley, CA, USA).

### Appendix A.2. Control Spheroids Formation and Hypoxia Induction

Control spheroids were cultured in an adapted version of our previously published microfluidic device [35]. Briefly, the wells are 700 × 700 × 700 µm^3^ instead of 500 × 500 × 500 µm^3^ and the channel contains 3 chambers of 21 wells instead of 5 chambers of 24 wells. The same method of device preparation and cell culture was applied; only cellular concentration was adjusted to 2 × 10^6^ cell/mL to avoid spheroids connection. In order to achieve the induction of hypoxia, control spheroids (<450 µm) were incubated for 24 h upon formation under a low O_2_ incubator at 24 h at 37 °C 86.8% N_2_, 2% O_2_, 5% CO_2_, 6.2% H_2_O.

### Appendix A.3. Image Analysis

#### Appendix A.3.1. Spheroid Diameter

Images of spheroids were taken using a brightfield microscope (EvosFLC, AMEFC4300R, Life Technologies) 2 days after seeding. A custom ImageJ (ImageJ, Fiji) batch processing analysis was implemented and used to analyze the diameter of the spheroids. Briefly, spheroid diameters are derived from spheroid area estimated in each image.

#### Appendix A.3.2. CAIX Localization and Quantification

Images of CAIX staining, obtained using a Zeiss fluorescence microscope with Axio-Vision 4.0 software, were analyzed using ImageJ. Briefly, CAIX positive fraction areas were estimated by intensity thresholding images, measuring the thresholded area, and dividing by the total cross-section area of the spheroids. CAIX staining intensity was quantify in control spheroids (small-normoxic and small-hypoxic) to assess induction of hypoxia in positive control. For each spheroid, an intensity histogram was taken, and the mean intensity was calculated. Then, pixel count strictly superior to the value of mean intensity was calculated and normalized by total pixel count of the same spheroid. Mean ratio of each N was calculated for small-normoxic and small-hypoxic spheroids of each cell line and compared using a 2-way ANOVA with a Šídák’s multiple comparisons test.

#### Appendix A.3.3. γH2AX Foci Quantification

Images of CAIX staining and γH2AX staining, obtained using a Zeiss fluorescence microscope with Axio-Vision 4.0 software, were analyzed using a custom macro on ImageJ Java 8. Briefly, in each image, the total nuclei area of the spheroid cross-section was estimated by thresholding the image in the DAPI channel. Then, the selection of the nuclei area was applied as a region of interest (ROI) in the γH2AX channel of the image, and the mean noise was measured. This value is used in the function “finding maxima” to retrieve and count every γH2AX foci strictly superior to the mean noise of the spheroid cross-section. In the red channel, CAIX-positive area was selected and applied as a ROI in the DAPI channel, before performing the previously described process for the CAIX-positive area only. γH2AX foci count in the CAIX-positive area was then normalized by nuclei area in the CAIX positive ROI. Finally, γH2AX foci count in the CAIX-negative area was calculated by subtracting γH2AX foci count in the CAIX-positive area from the total and normalized by total area minus CAIX-positive nuclei area.
