# Supplementary material for: Hypoxic Jumbo Spheroids On-A-Chip (HOnAChip): Insights into Treatment Efficacy
Source: Cancers (Basel). 2021 Aug 11;13(16):4046. doi: 10.3390/cancers13164046 (PMC8394550; doi:10.3390/cancers13164046)

Supplementary Materials: Hypoxic Jumbo Spheroids On-A-Chip (HOnAChip): Insights into Treatment Efficacy

Figure S1. Uncropped Western Blot Figures.

STS117_N1_Actin
Chemiluminescence

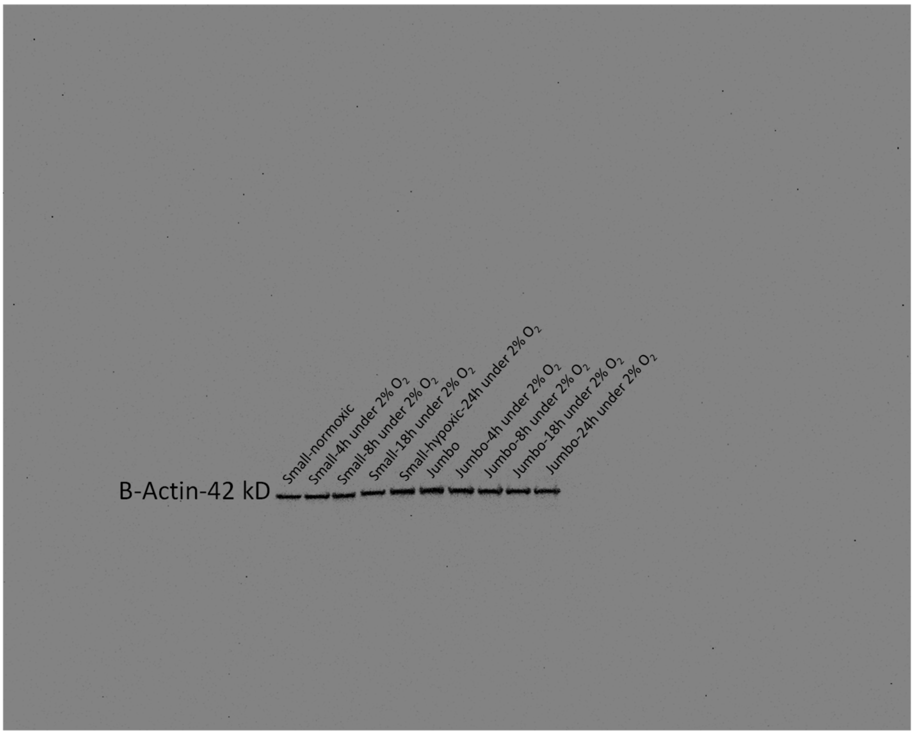

STS117_N1_Actin
Composite

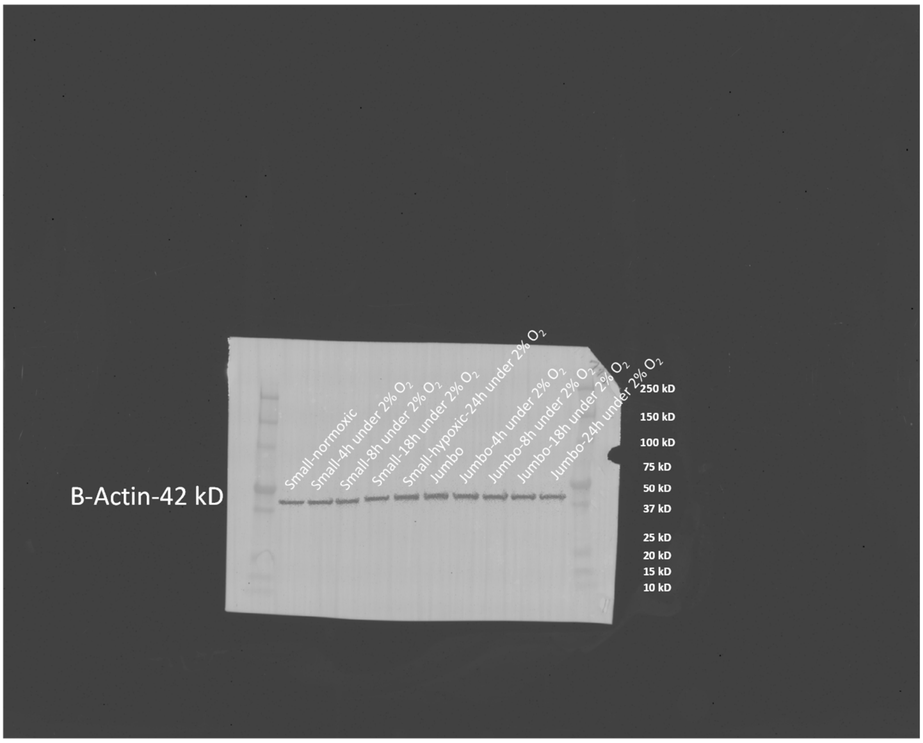

STS117_N1_CAIX
Chemiluminescence

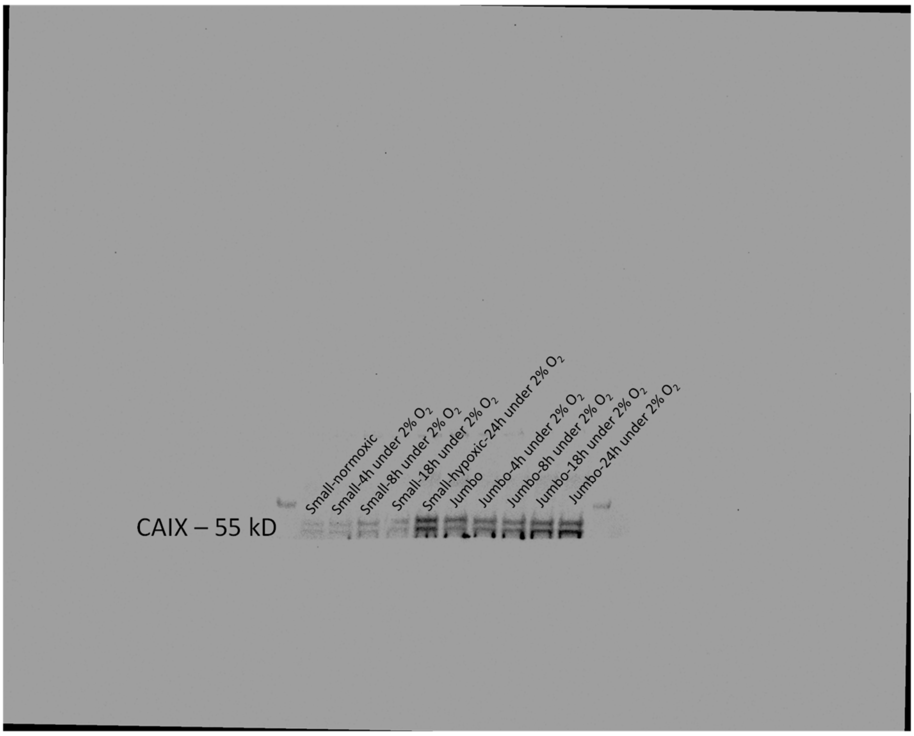

STS117_N1_CAIX
Composite

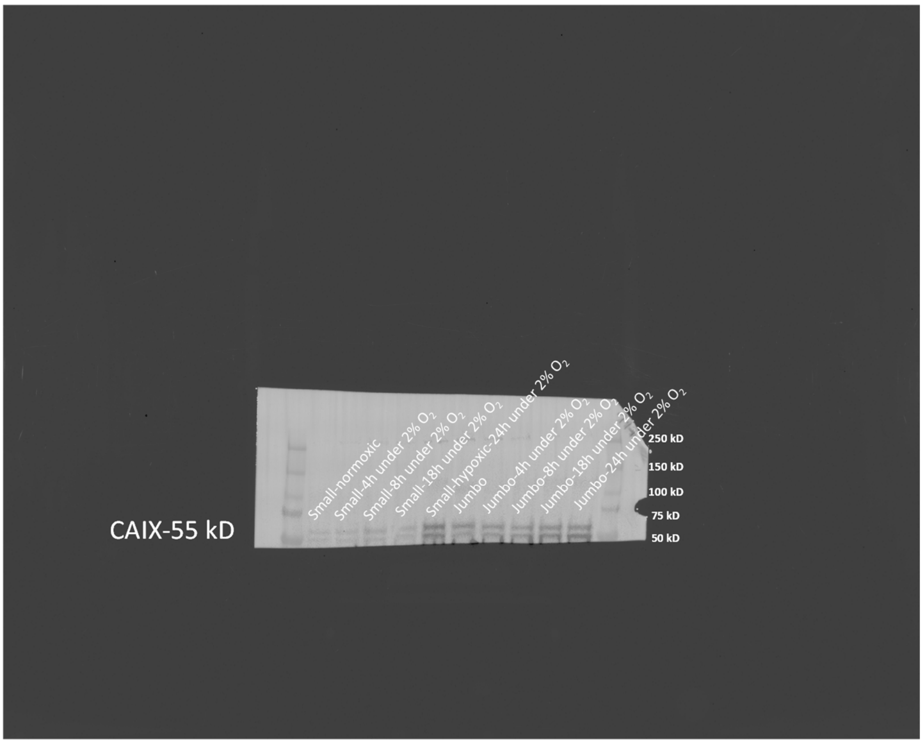

STS117_N1_HIF1-α
Chemiluminescence

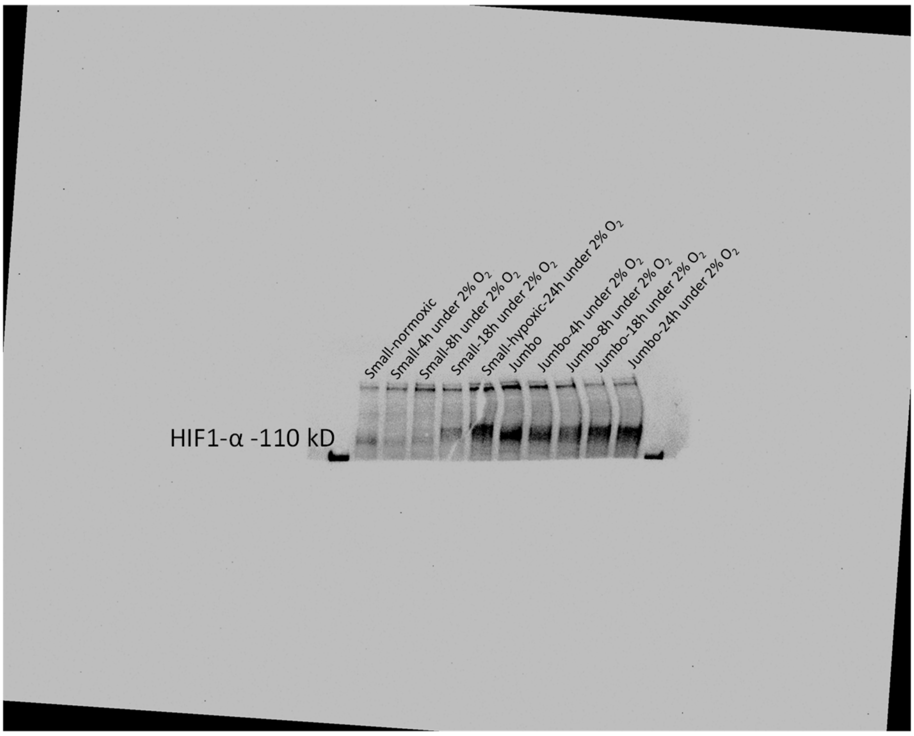

STS117_N1_HIF1-α
Composite

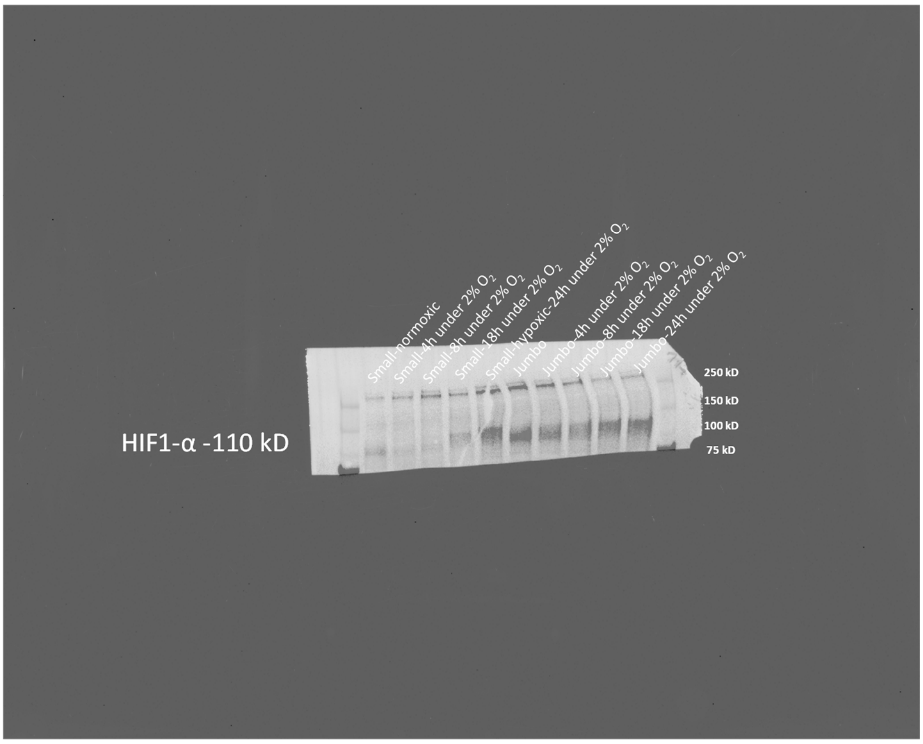

SK-LMS-1_N1_Actin
Chemiluminescence

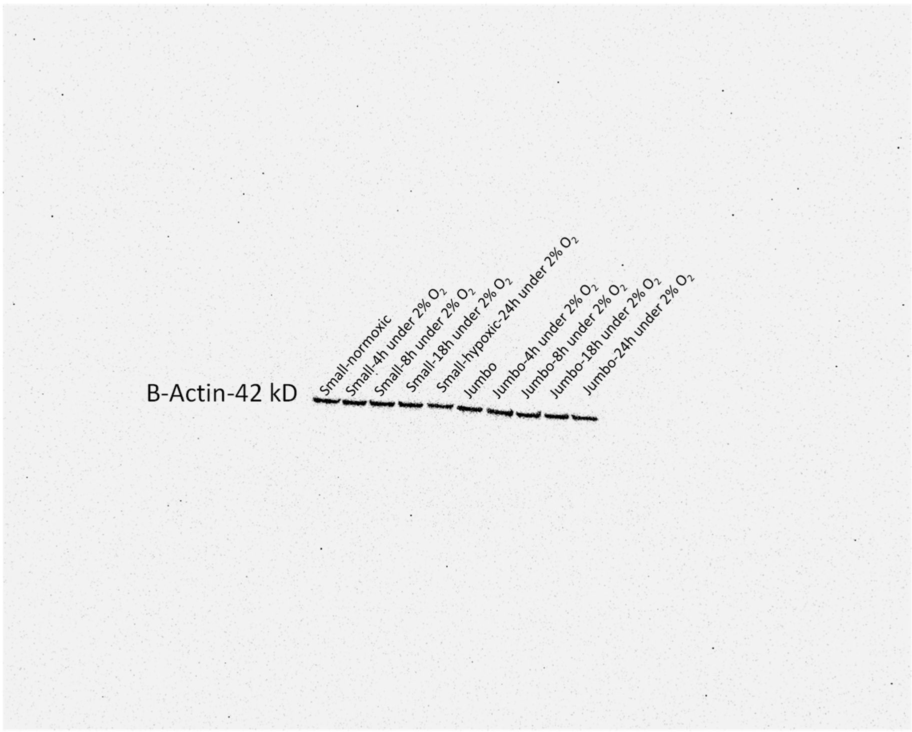

SK-LMS-1_N1_Actin
Composite

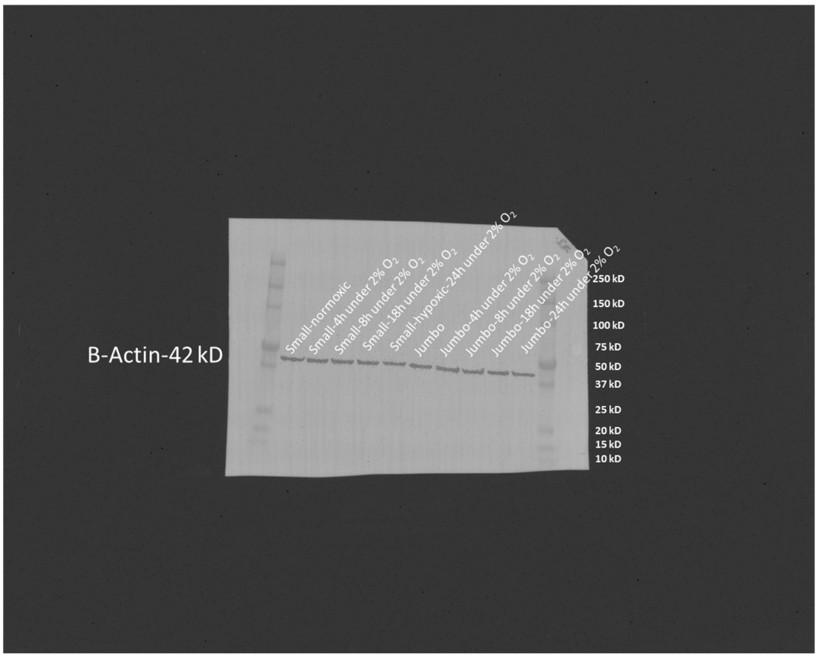

SK-LMS-1_N1_CAIX
Chemiluminescence

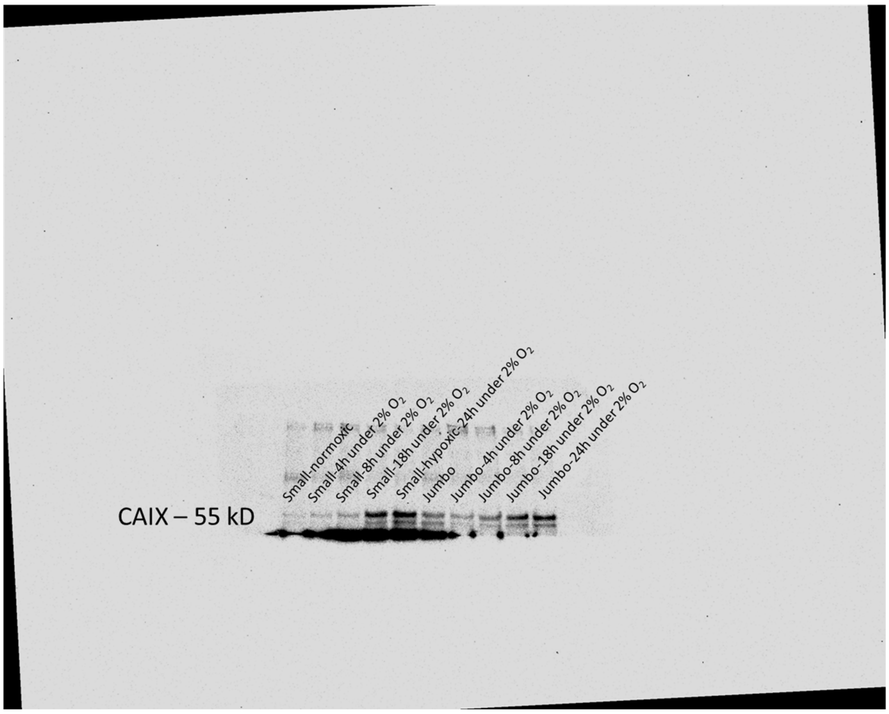

SK-LMS-1_N1_CAIX
Composite

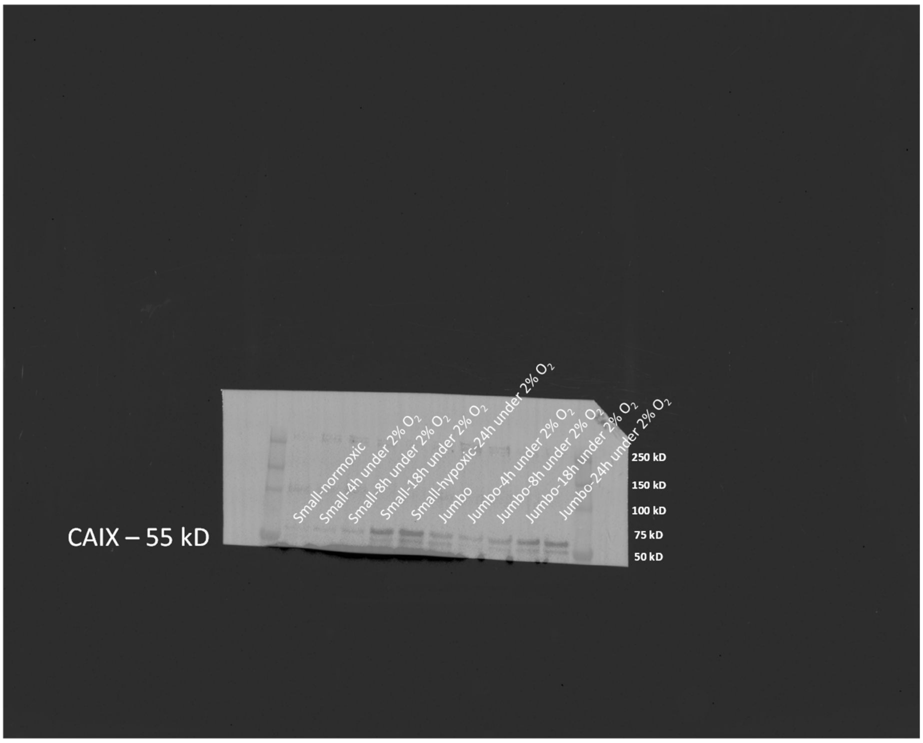

SK-LMS-1_N1_HIF1-α
Chemiluminescence

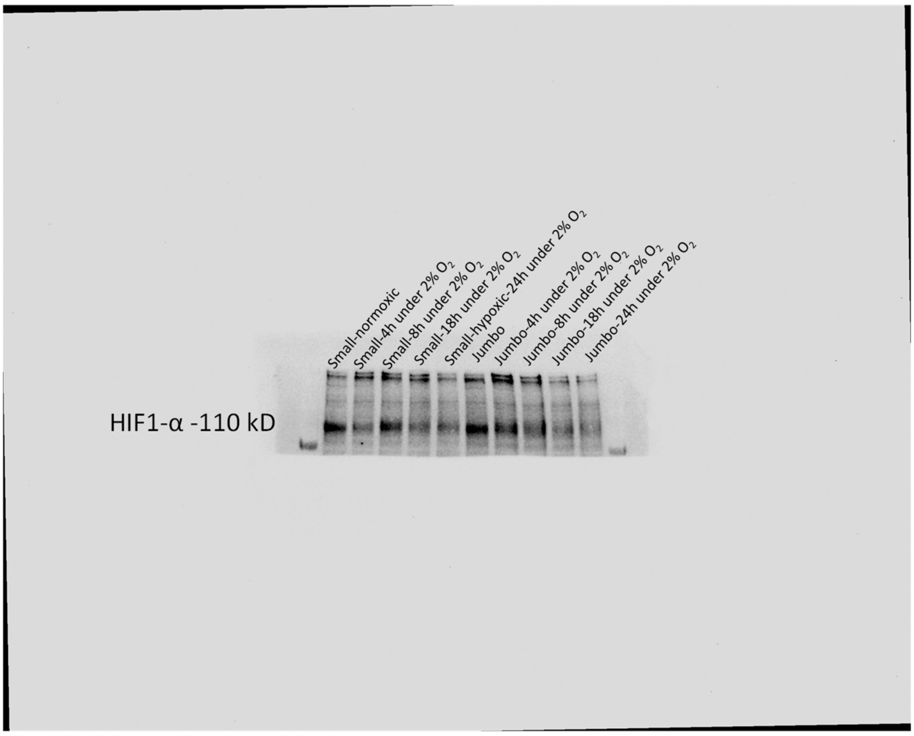

SK-LMS-1_N1_HIF1-α
Composite

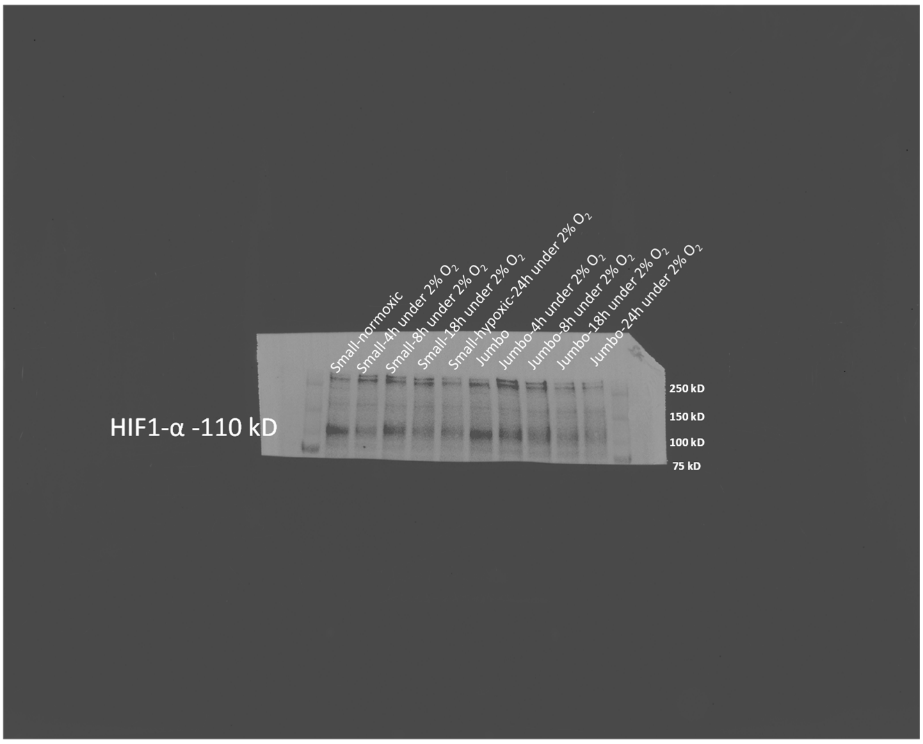

STS117_N2_Actin
Chemiluminescence

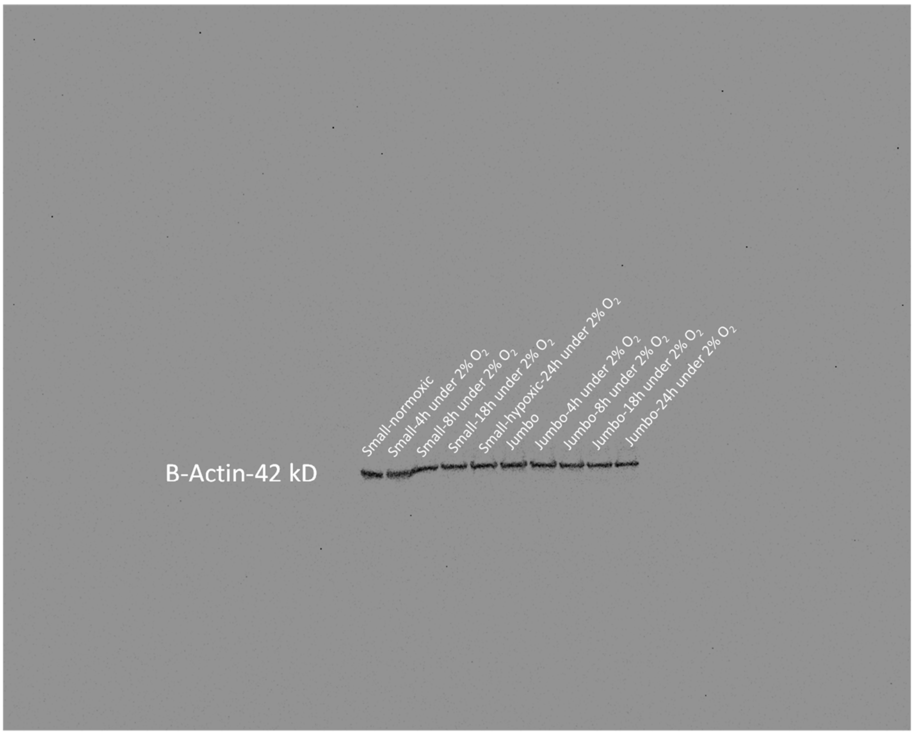

STS117_N2_Actin
Composite

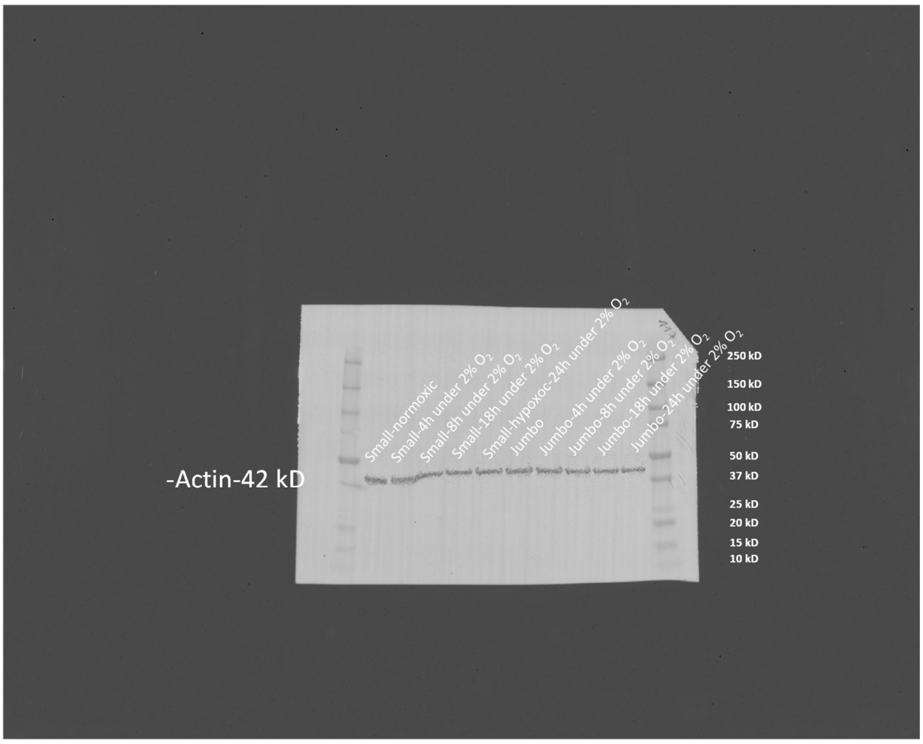

STS117_N2_CAIX
Chemiluminescence

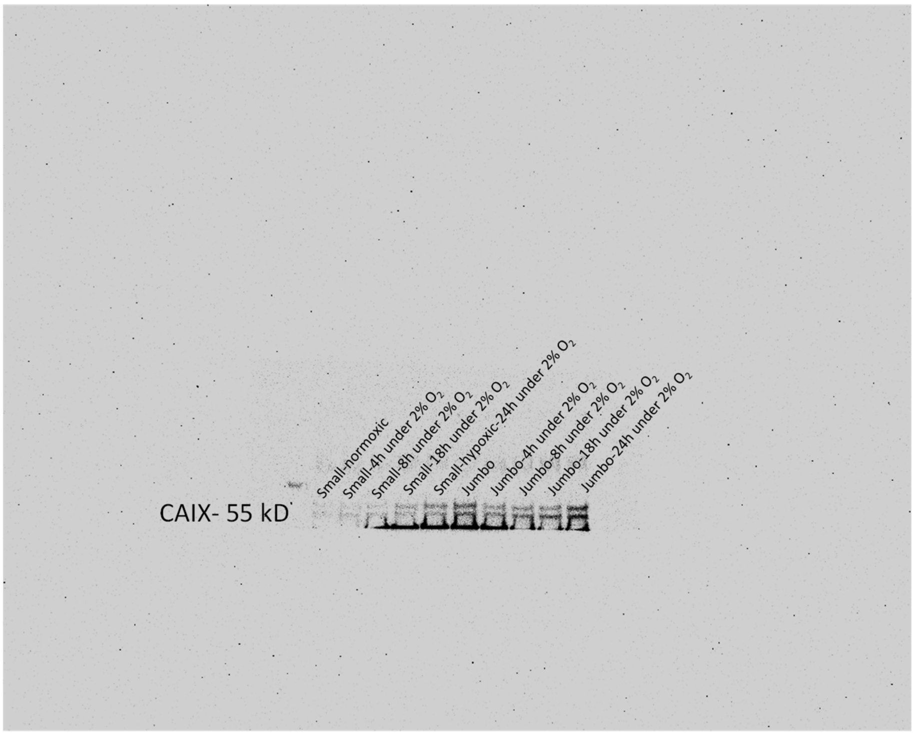

STS117_N2_CAIX
Composite

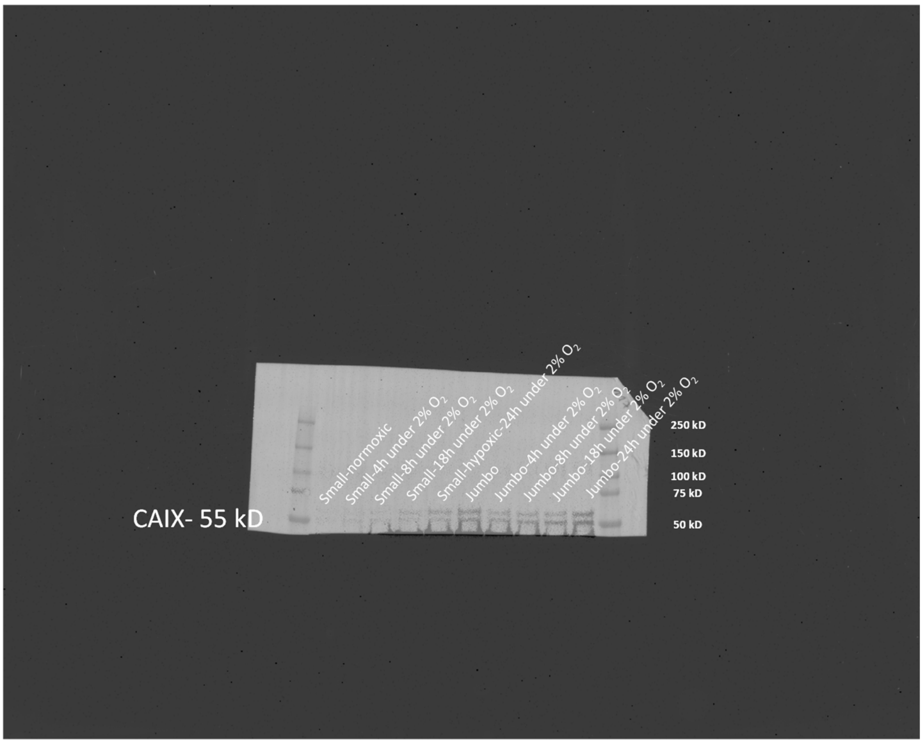

STS117_N2_HIF1-α
Chemiluminescence

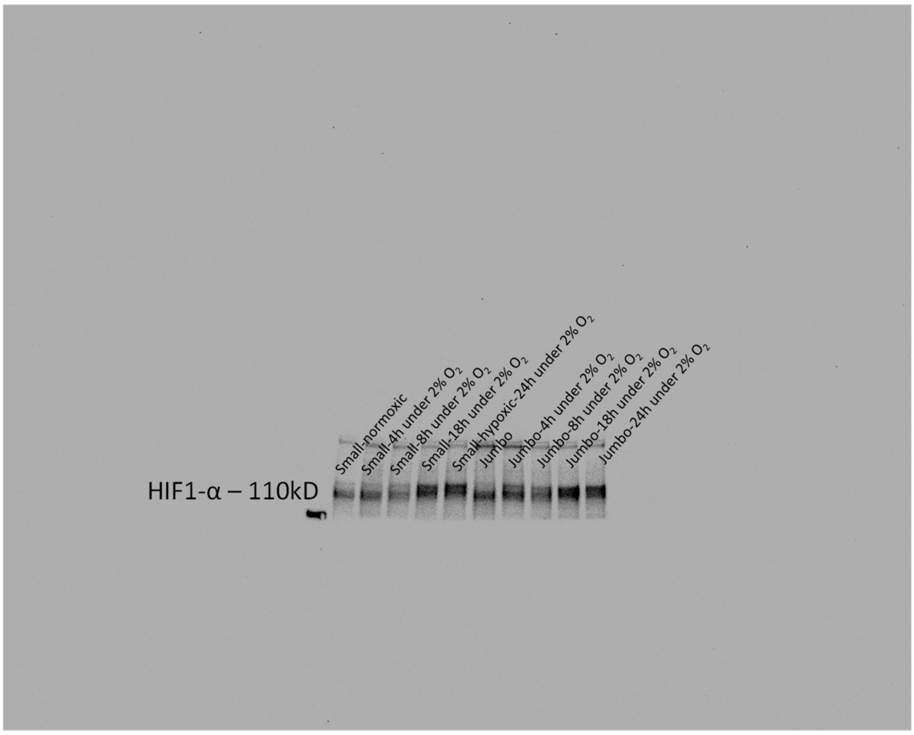

STS117_N2_HIF1-α
Composite

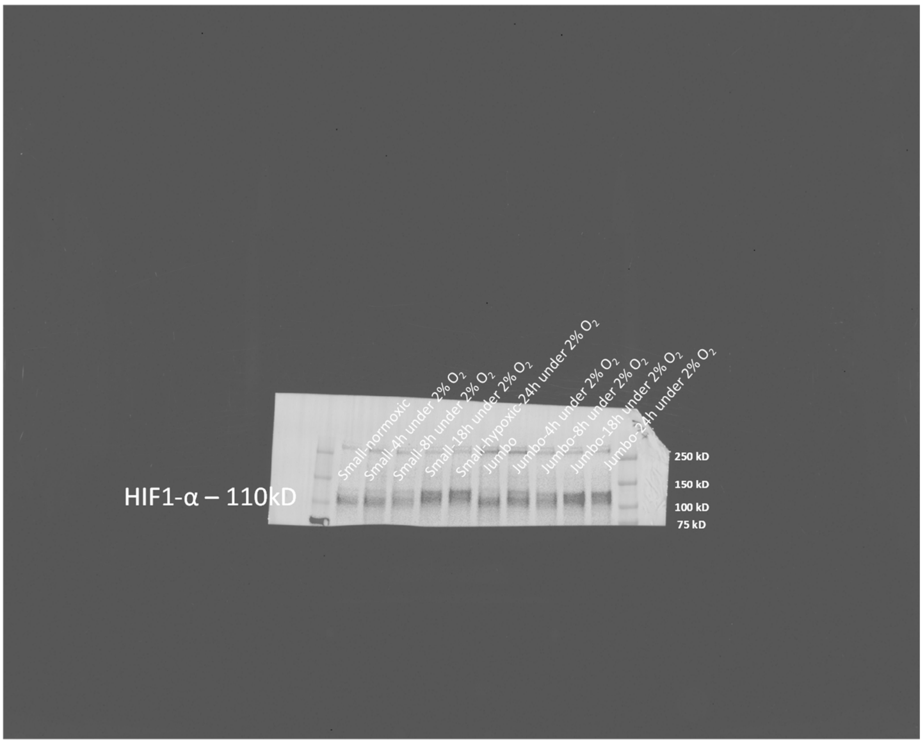

SK-LMS-1_N2_Actin
Chemiluminescence

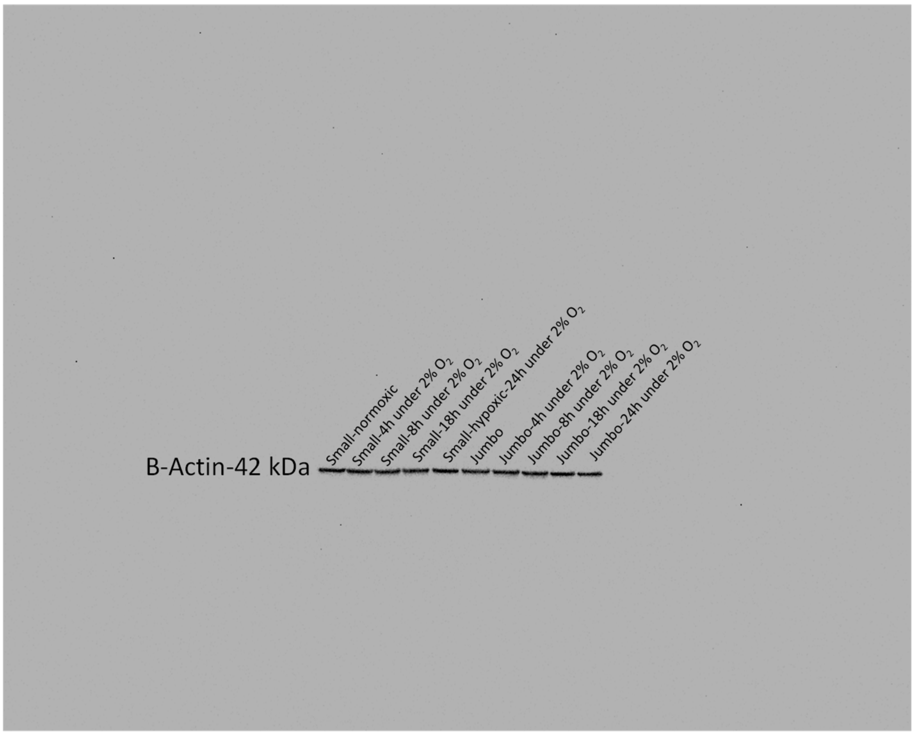

SK-LMS-1_N2_Actin
Composite

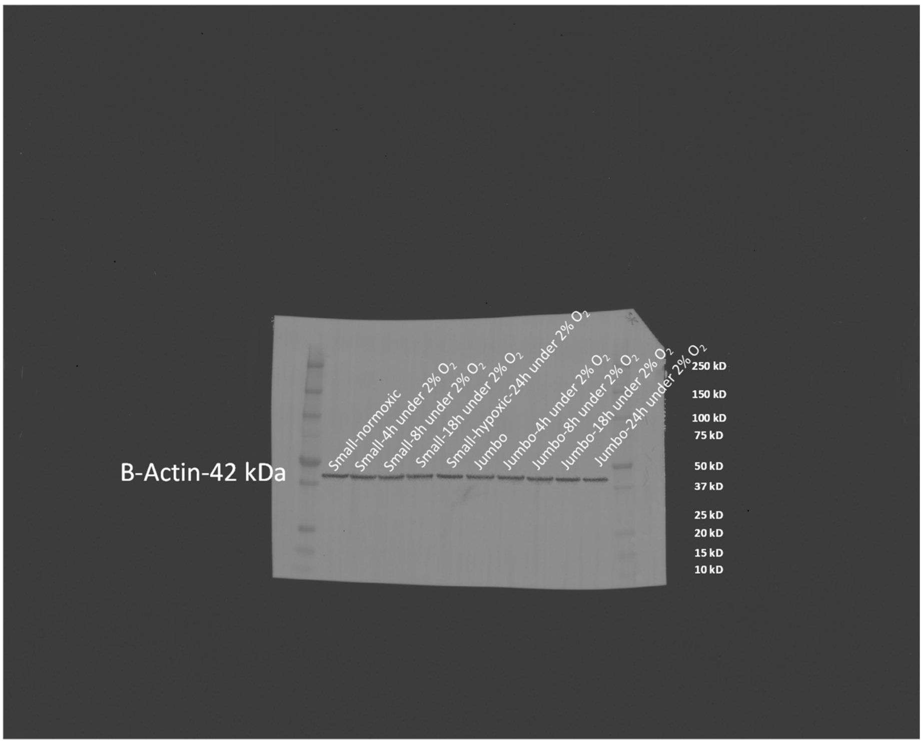

SK-LMS-1_N2_CAIX
Chemiluminescence

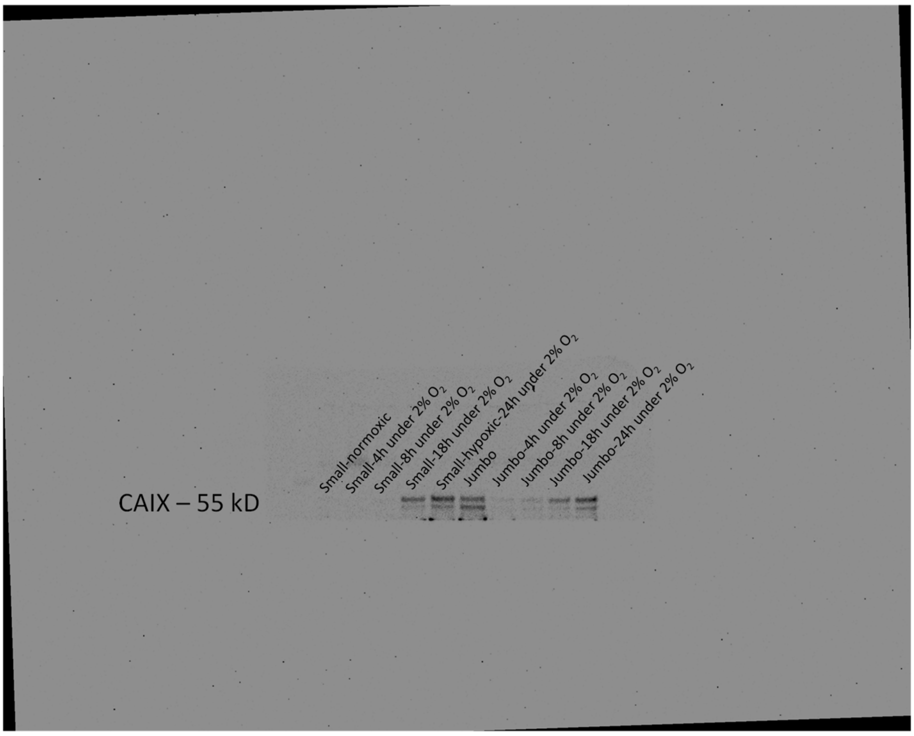

SK-LMS-1_N2_CAIX
Composite

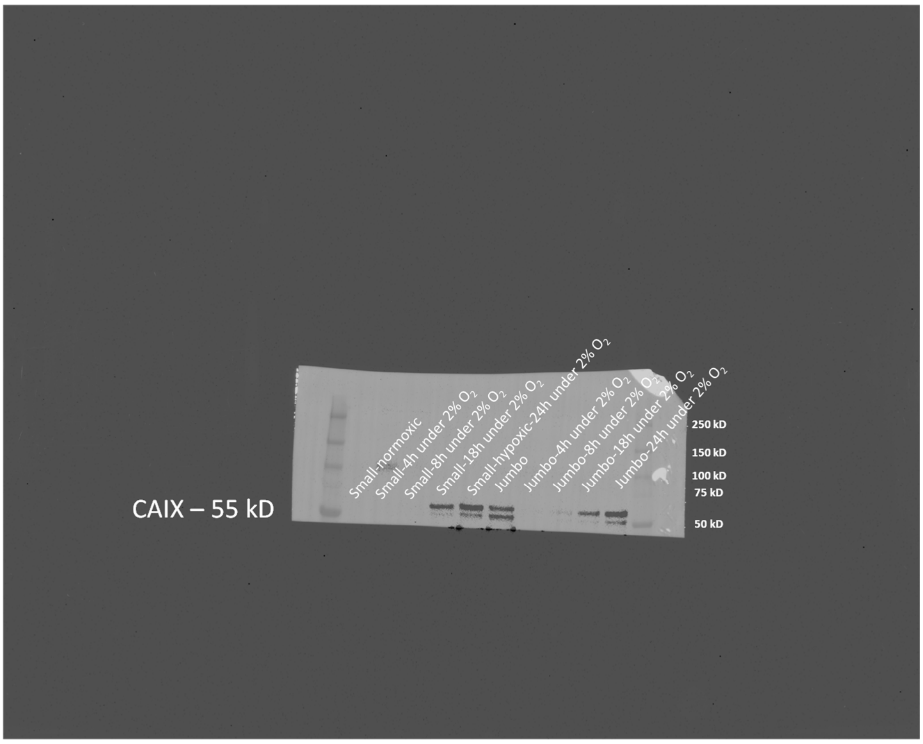

SK-LMS-1_N2_HIF1-α
Chemiluminescence

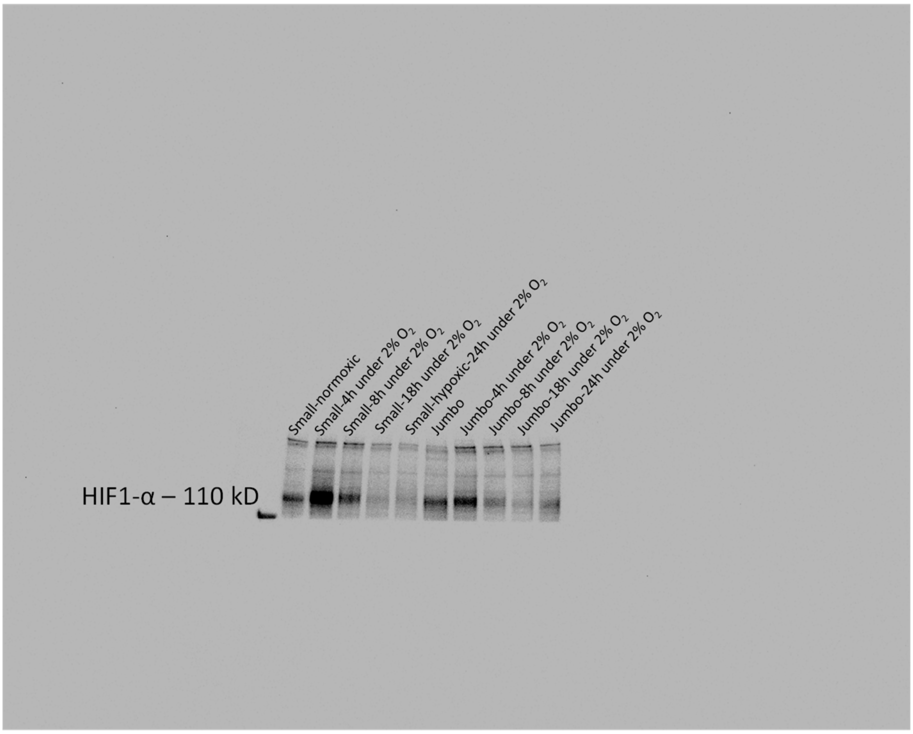

SK-LMS-1_N2_HIF1-α
Composite

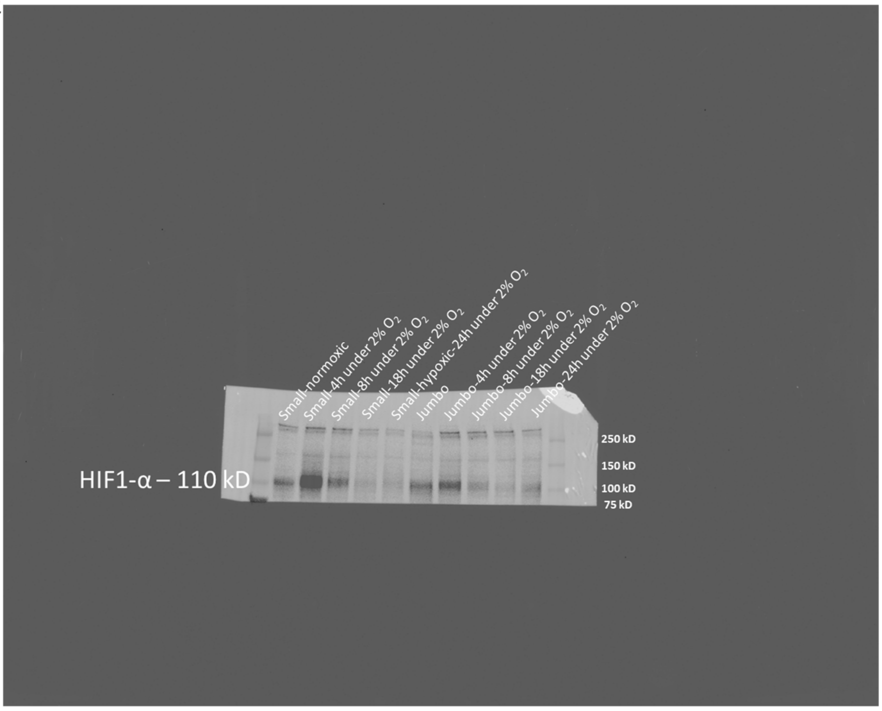

STS117_N3_Actin
Chemiluminescence

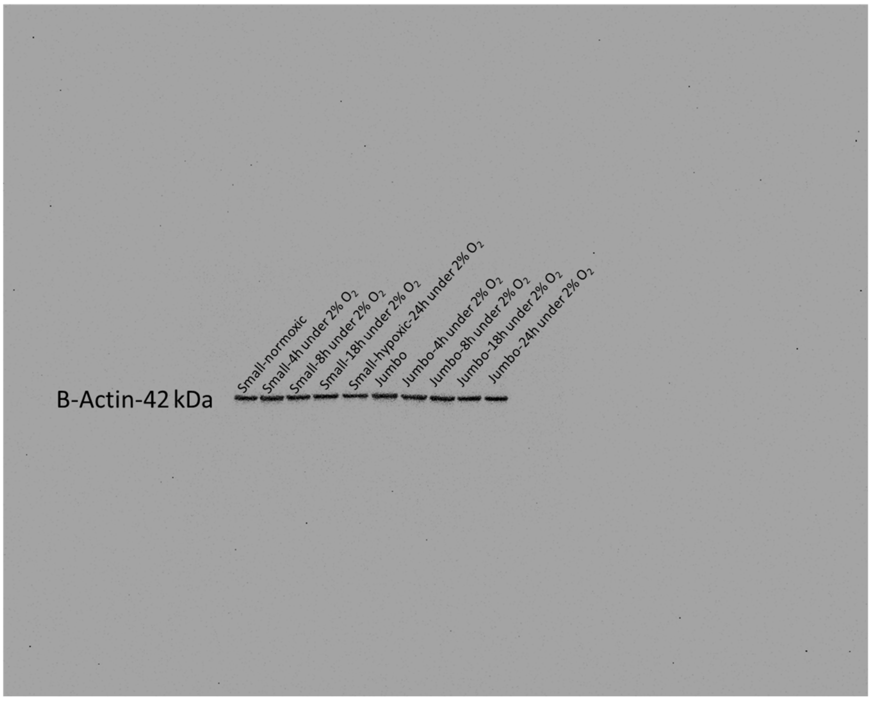

STS117_N3_Actin
Composite

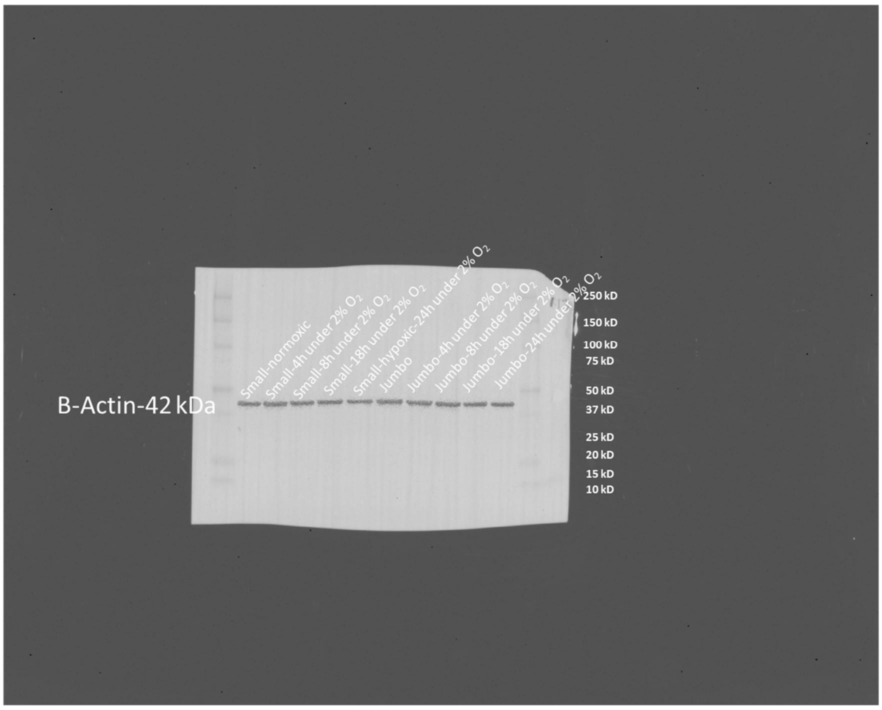

STS117_N3_CAIX
Chemiluminescence

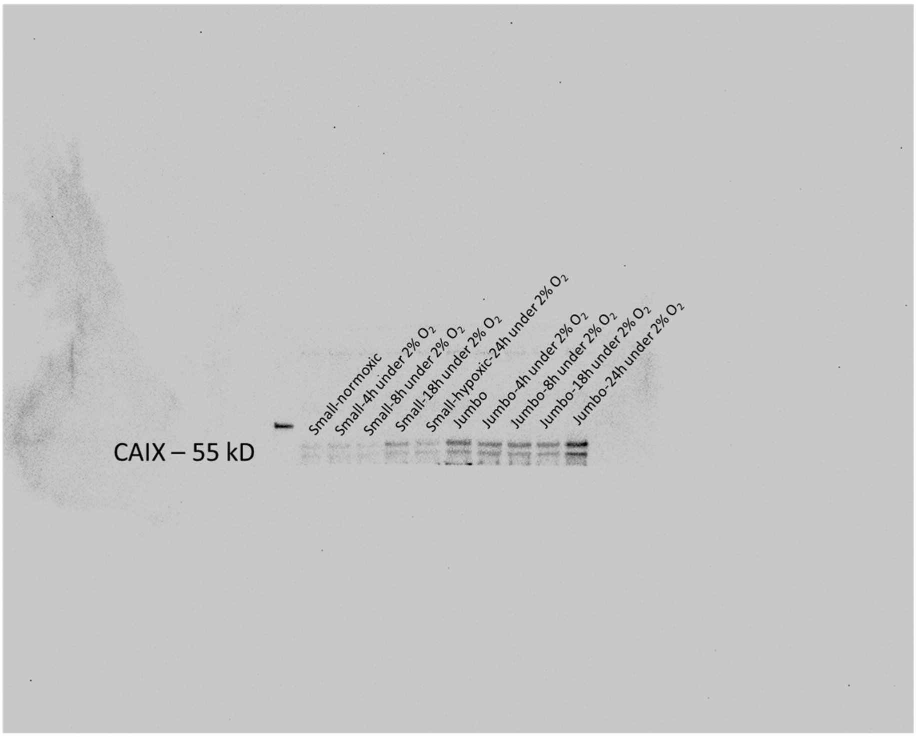

STS117_N3_CAIX
Composite

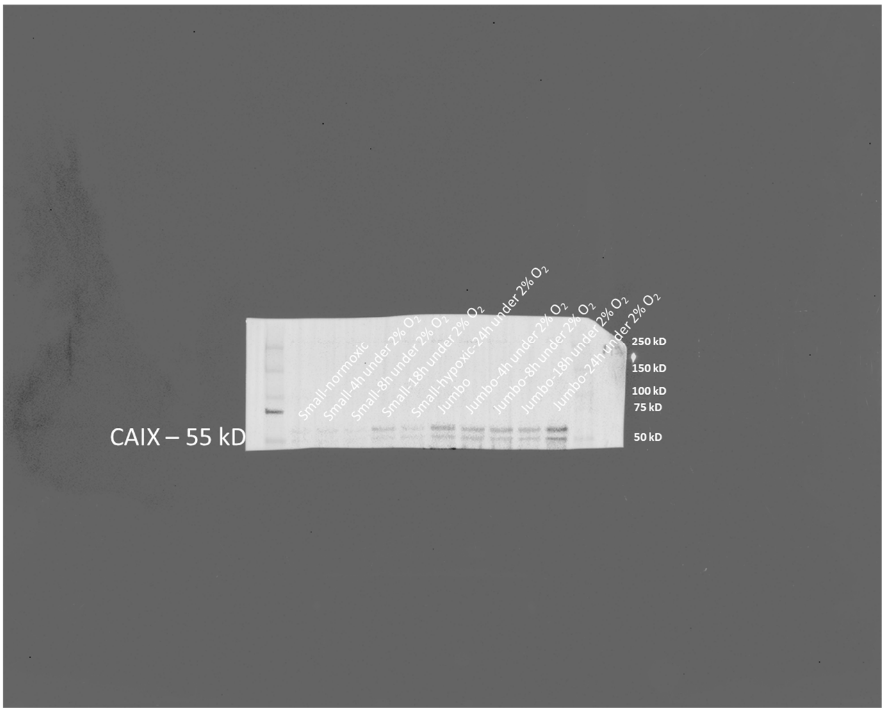

STS117_N3_HIF1-α
Chemiluminescence

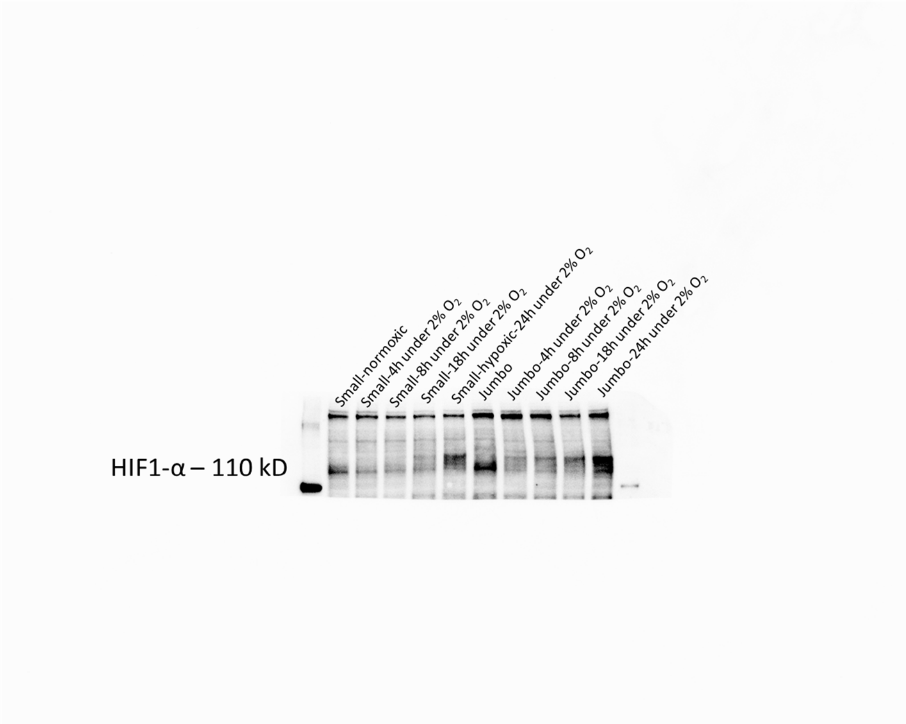

STS117_N3_HIF1-α
Composite

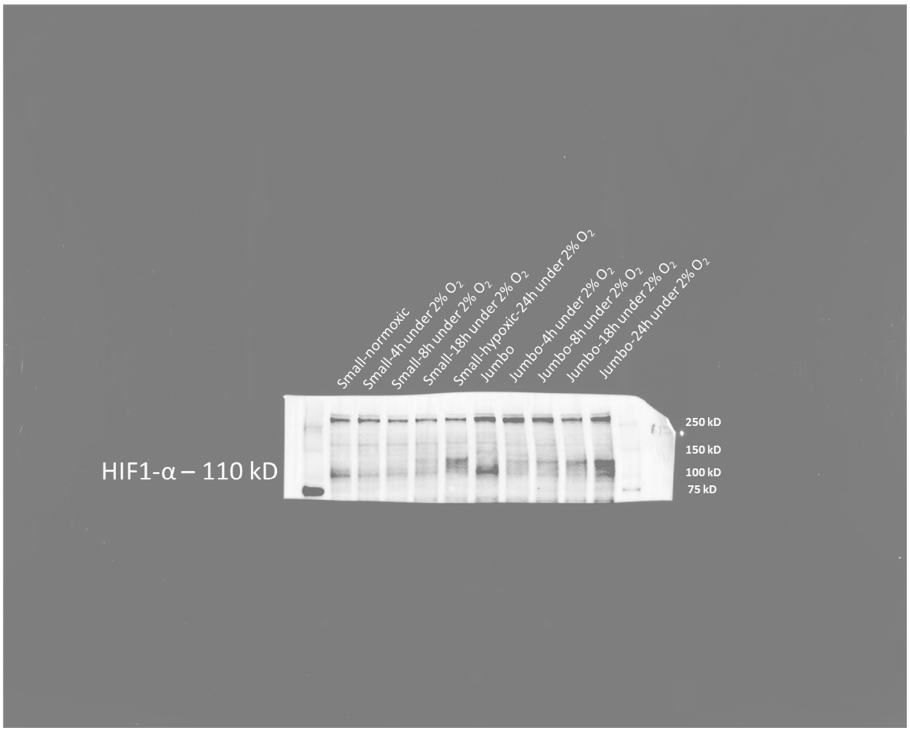

SK-LMS-1_N3_Actin
Chemiluminescence

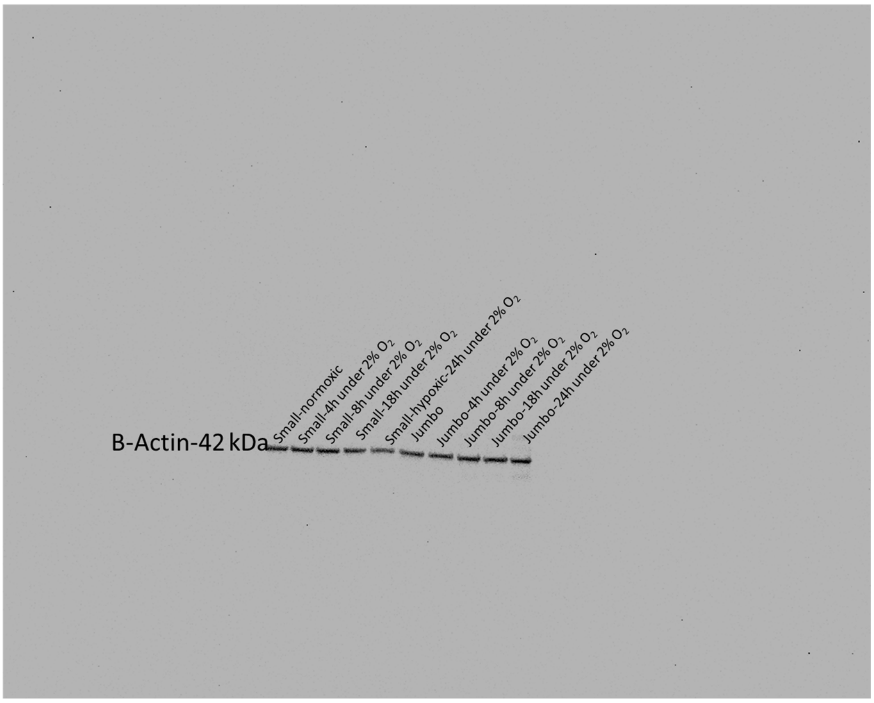

SK-LMS-1_N3_Actin
Composite

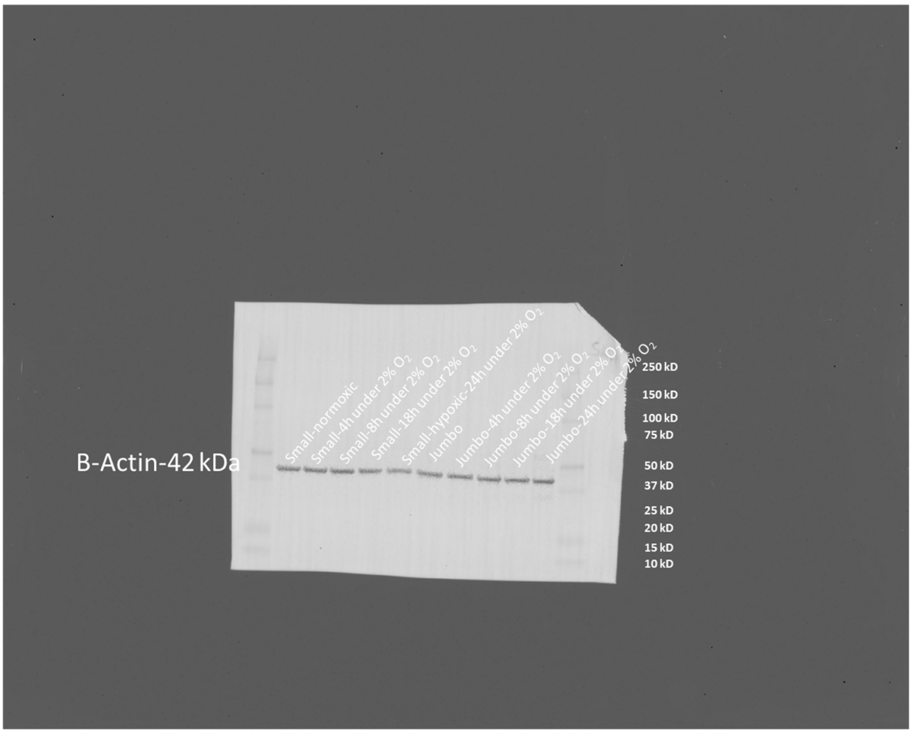

SK-LMS-1_N3_CAIX
Chemiluminescence

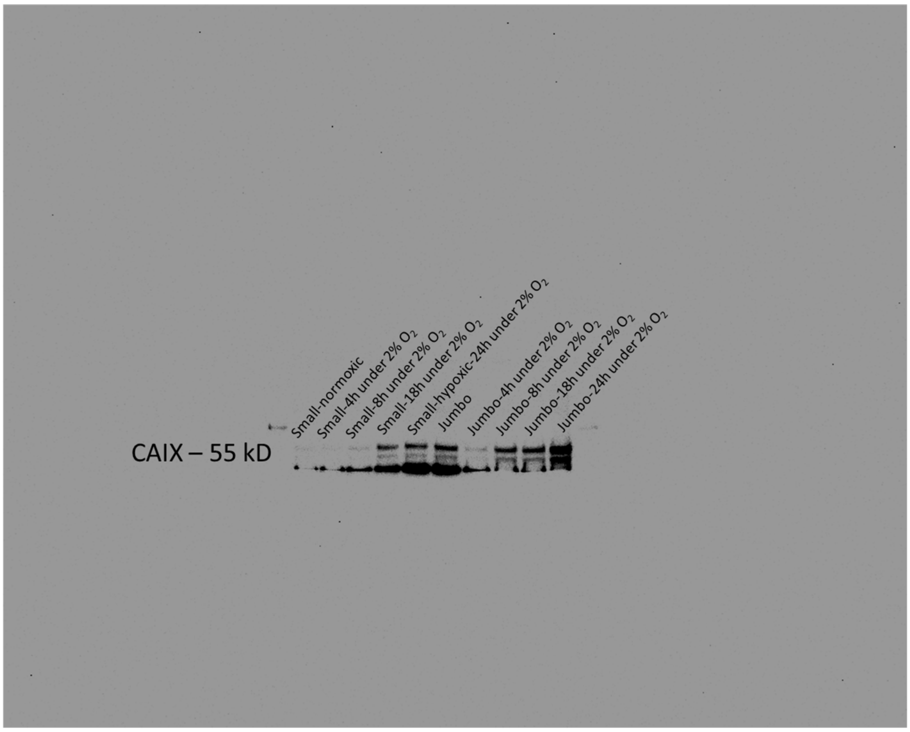

SK-LMS-1_N3_CAIX
Composite

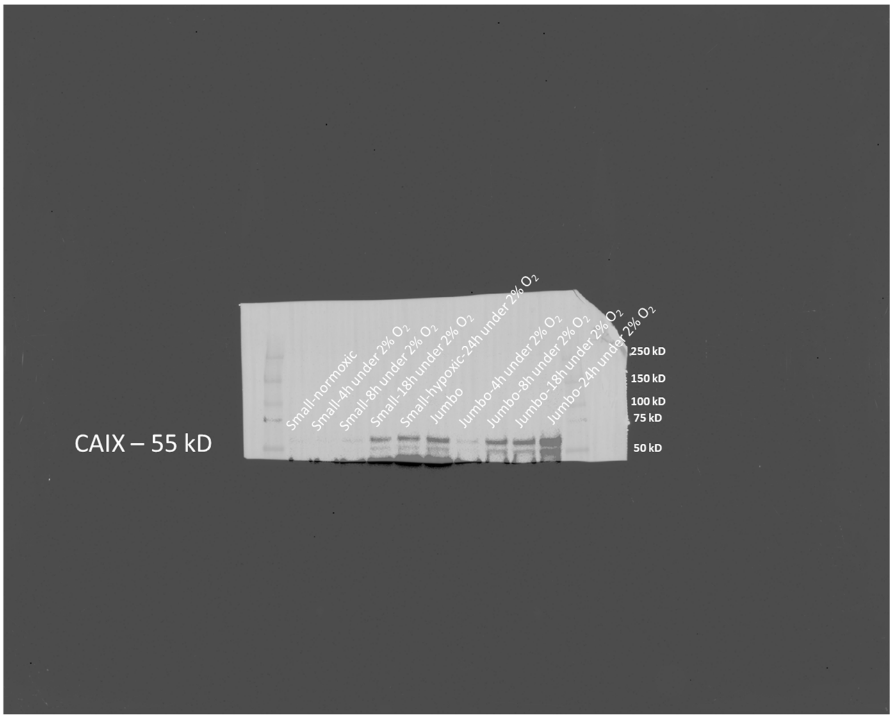

SK-LMS-1_N3_HIF1-α
Chemiluminescence

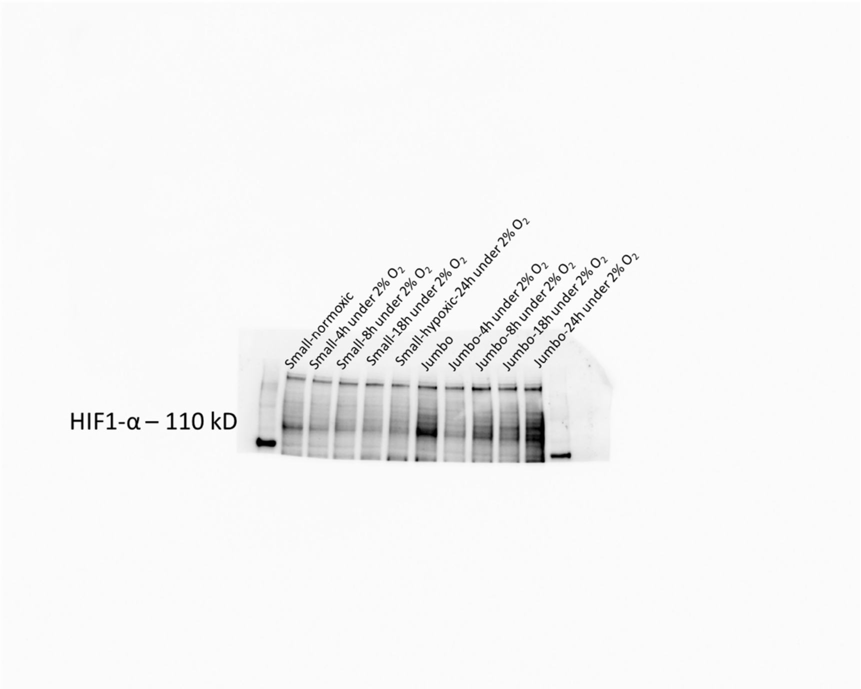

SK-LMS-1_N3_HIF1-α
Composite

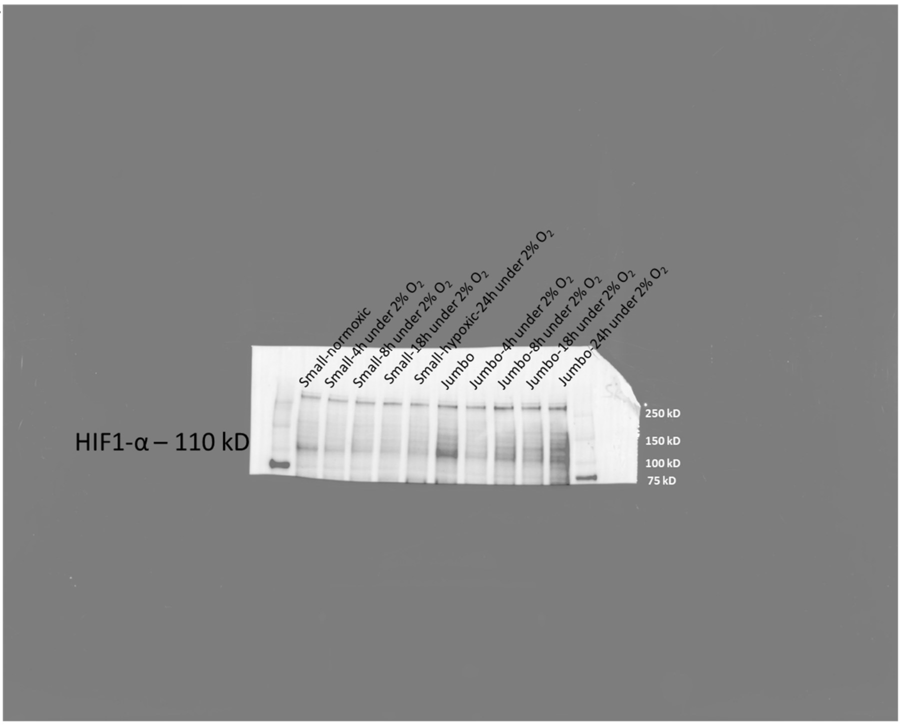

Supplement: Supplementary file 1 [file cancers-13-04046-s001.zip › cancers-1312972-supplementary.pdf]
